# Mutation and apoptosis are well-coordinated for protecting against DNA damage-inducing toxicity in *Drosophila*

**DOI:** 10.1186/s41021-023-00267-4

**Published:** 2023-03-23

**Authors:** Megumi Toyoshima-Sasatani, Fumika Imura, Yuko Hamatake, Akihiro Fukunaga, Tomoe Negishi

**Affiliations:** 1 Graduate School of Medicine, Dentistry, and Pharmaceutical Sciences, Okayama University, Okayama Tsushima, 700-8530 Japan; 2 Research Institute for Radiation Biology and Medicine, Hiroshima University, Kasumi, Minami-ku, Hiroshima, 734-8553 Japan; 3 School of Nursing, Osaka City University, Abeno-Ku, Osaka, 545-0051 Japan

**Keywords:** *Drosophila*, Apoptosis, Mutation, Larval wing disc, X-ray, Ultraviolet, Alkylating agents, Tobacco smoke, Acridine orange, BrdU

## Abstract

**Background:**

Apoptotic cell death is an important survival system for multicellular organisms because it removes damaged cells. Mutation is also a survival method for dealing with damaged cells in multicellular and also unicellular organisms, when DNA lesions are not removed. However, to the best of our knowledge, no reports have comprehensively explored the direct relationship between apoptosis and somatic cell mutations induced by various mutagenic factors.

**Results:**

Mutation was examined by the wing-spot test, which is used to detect somatic cell mutations, including chromosomal recombination. Apoptosis was observed in the wing discs by acridine orange staining in situ. After treatment with chemical mutagens, ultraviolet light (UV), and X-ray, both the apoptotic frequency and mutagenic activity increased in a dose-dependent manner at non-toxic doses. When we used DNA repair-deficient *Drosophila* strains, the correlation coefficient of the relationship between apoptosis and mutagenicity, differed from that of the wild-type. To explore how apoptosis affects the behavior of mutated cells, we determined the spot size, *i.e.,* the number of mutated cells in a spot. In parallel with an increase in apoptosis, the spot size increased with MNU or X-ray treatment dose-dependently; however, this increase was not seen with UV irradiation. In addition, BrdU incorporation, an indicator of cell proliferation, in the wing discs was suppressed at 6 h, with peak at 12 h post-treatment with X-ray, and that it started to increase again at 24 h; however, this was not seen with UV irradiation.

**Conclusion:**

Damage-induced apoptosis and mutation might be coordinated with each other, and the frequency of apoptosis and mutagenicity are balanced depending on the type of DNA damage. From the data of the spot size and BrdU incorporation, it is possible that mutated cells replace apoptotic cells due to their high frequency of cell division, resulting in enlargement of the spot size after MNU or X-ray treatment. We consider that the induction of mutation, apoptosis, and/or cell growth varies in multi-cellular organisms depending on the type of the mutagens, and that their balance and coordination have an important function to counter DNA damage for the survival of the organism.

**Supplementary Information:**

The online version contains supplementary material available at 10.1186/s41021-023-00267-4.

## Introduction

There are numerous DNA-damaging factors in the environment of organisms, including substances in food, air and water, and physical factors, such as ultraviolet (UV) light and X-rays. As the primary defense system against the many accidental lesions that occur continually in DNA, organism possess multiple DNA repair pathways to maintain the genetic stability for its survival. Under normal circumstances, damage that occurs in DNA is efficiently repaired by native DNA repair systems according to each type of damage [1–5]. When the DNA damage cannot be repaired, the cells seem to either survive by compensating for the lesion, or undergo cell death. For multicellular organisms, apoptotic cell death is considered to be an important repair system, because an organism can survive if the damaged cells are removed. This is known as apoptotic repair [6, 7]. Mutation is also a survival method for dealing with damaged cells when DNA lesions that cause replication fork stalling are not removed. The balance between apoptosis and mutation appears to play an important role in mutagenesis leading to carcinogenesis in multicellular eukaryotes, and the efficacy of DNA repair is thought to be a critical factor in this process.

*Drosophila melanogaster* is widely used as a model organism to examine the biological events including DNA-damage response, because many investigations with this insect were performed more than one century and it has been proved that it has many orthologue genes to human and other mammals [8–11]. *Drosophila* undergoes a complete metamorphosis from egg, larva, pupa to adult fly. As frequent cell division occurs only at the stage of embryo and at the cells composing imaginal discs, which are larval organs developing to adult tissues such as wings, eyes, legs and so on [12]. The cells composing adult flies do not proliferate except for stem cells. The cell division during development is stopped at pupal stage and then metamorphosis occurs due to apoptosis known as programed cell death, which occurs in the no relationship with DNA damage by mutagens, to develop from pupa to adult fly [13]. To observe the mutagenic process induced by DNA damaging factors, the treatment with them is restricted at either the embryonic or larval stage. The *Drosophila* wing-spot test [14] is known to be a short-term, sensitive, and non-mammalian in vivo test method for examining the effects of genotoxins and mutagens in the environment. It relies on the induction of genetic damage in dividing wing disc cells in *Drosophila* third instar larvae, which can be easily observed on the adult wings as mutant wing spots. In this mutation test, we can detect the somatic cell mutations including chromosomal recombination, non-disjunction, segmental chromosomal deletion and gene mutations. Furthermore, the behavior of the mutated cells can be examined by counting the number of mutated cells contained in a spot as the spot size, which indicates the frequency of mutated cell division [14]. In addition, *Drosophila*-based research has the advantage of using many kinds of genetic strains such as DNA repair-deficient strains and oxidative damage sensitive strains. The use of DNA repair-deficient strains will allow us to study the effect of DNA repair systems on mutagenesis and apoptosis after the treatment of DNA damaging factors. So far, we have been proved the mutagenic activities and DNA damage induced by various factors such as X-ray, sunlight, UV light and many chemical mutagenic carcinogens, using the *Drosophila* wing-spot test.

It is well-documented that apoptosis, as a programmed cell death process, plays important roles in the development of multicellular organisms including *Drosophila* [13]. Moreover, the knowledge is accumulated that apoptosis is also induced by DNA damage [15, 16]. Recently, Baonza et al. reviewed about the genetic regulation leading to either apoptosis or non-apoptosis corresponding to the DNA damage induced by ionizing radiation in *Drosophila* [17]. However, to the best of our knowledge, no reports have comprehensively explored the direct relationship between apoptosis and somatic cell mutations induced by DNA damage. We investigated the relationship between apoptosis and mutation using *Drosophila melanogaster*, as apoptosis and mutation can both be examined in the same organ, *i.e.,* larval wing discs.

The detection of apoptosis is performed by various methods that are applicable to any organism, including the detection of morphological changes, *i.e.,* cell shrinkage, chromatin condensation and nuclear fragmentation, the detection of DNA fragmentation by the TdT-mediated dUTP-biotin nick end-labeling (TUNEL) assay targeting 3’-OH ends following DNA breaks, the DNA ladder assay showing inter-nucleosomal fragmentation, the comet assay, immunohistochemistry detecting apoptosis-related substances, such as p53, annexin V and caspase 3 [18, 19], and the acridine orange (AO)-staining method [20, 21]. Arama and Steller have reported the use of a TUNEL assay and AO staining to detect apoptosis in *Drosophila* embryos and adult male gonads [22]. In the present study, we examined several methods, and selected the AO-staining method to be the most appropriate for the detection of apoptosis in larval wing discs, which are used for the detection of somatic cell mutations in the wing-spot test [14].

**Fig. 1:**
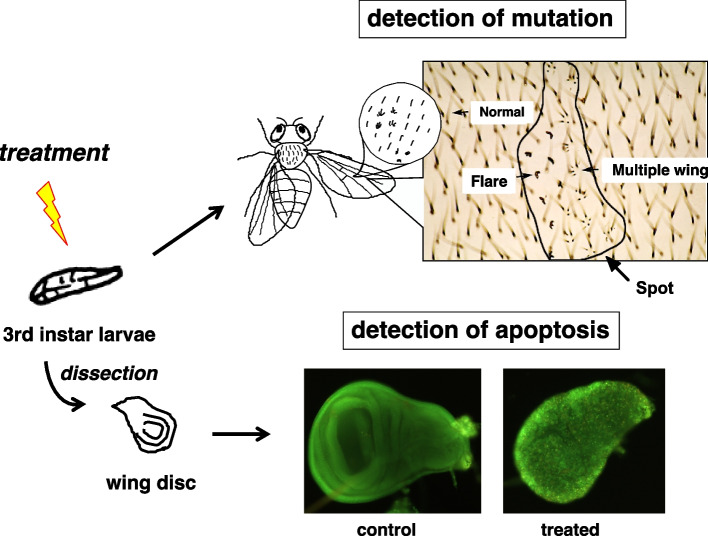
The experimental scheme of this study. *Drosophila* third instar larvae were treated with various factors. Wing discs were dissected from a portion of the larvae at different time points post-treatment, and apoptosis was scored. The remaining larvae were kept at 25 °C until they reached adulthood, then the colonies consisting of mutated cells were identified as spots on the wings

To investigate the correlation between apoptosis and mutation, we observed the apoptosis and mutation induced by several chemical mutagens, UV light, and X-ray according to the scheme shown in Fig. 1. Moreover, it is well known that DNA repair system plays important roles in mutagenic process [1] and also apoptotic process is regulated by DNA repair [16, 17]. Therefore, we examined how the DNA repair pathway influences apoptosis and mutation, and how apoptosis affects the behavior of mutated cells.

## Materials and methods

### *Drosophila* strains

To detect somatic cell mutations, the wing-spot test was performed using repair-proficient strains (*y; mwh j v* and *y; Dp(1;3)sc*^*J4*^*, y*^+^ *flr/TM1, Mè ri sbd*^*2*^*)* and repair-deficient strains (*cn*^*35*^* mus201*^*D1*^*; mwh j v* and *cn*^*35*^* mus201*^*D1*^*; flr*^*3*^*/TM2, Ser*) as described below. The *mus201*^*D1*^ mutants (abbreviated as *mus201*) [23] are a set of analogs of the excision-defective XPG cell lines, which were gifts from Dr. H. Ryo (Osaka University, Suita, Japan). The genes *mwh* and *flr* are recessive markers of wing hair, and homozygotes of these genes show multiple wing hairs and flare hairs, respectively. The *sc z*^*1*^* w*^+*(TE)*^*/C(1)DX, y f* strain (abbreviated as *mei*^+^), which is a repair-proficient strain, the *sc z*^*1*^* w*^+*(TE)*^* mei-9*^*a*^*/C(1)DX, y f* strain (abbreviated as *mei-9*) consisting of nucleotide excision repair (NER)-deficient males (XPF homologue), and the *sc z*^*1*^* w*^+*(TE)*^*mei-41*^*D5*^*/C(1)DX, y f* strain (abbreviated as *mei-41*) consisting of ataxia telangiectasia and Rad3-related protein (ATR)-deficient males that are deficient in post-replication repair were kindly provided by Dr. H. Frei and Dr. U. Graf (University of Zurich, Schwerzenbach, Switzerland). The *mei-9* mutant was established by Boyd et al*.* as a mutant defective in the excision of UV-induced pyrimidine dimers [24]. The *mei-41* mutant was characterized to be post-replication repair-deficient by Boyd and Setlow [25]. The *mei-41* gene has been identified as an ATR ortholog gene [26, 27]. *C(1)DX* is an attached X chromosome with wild-type alleles of *mei-9*^*a*^ and *mei-41*^*D5*^. A urate-null strain carrying the mutant allele *ma-l* that is sensitive to oxidative damage due to the lack of xanthine dehydrogenase activity, and thus has a urate-null phenotype [28], and Oregon-R, which was used as a control wild-type strain, were kindly provided by Dr. H. Ryo (Osaka University, Suita, Japan). These genotypes have been described by Lindsley and Zimm [29].

### Treatment of *Drosophila* larvae

*Drosophila* third instar larvae were treated in a plastic petri dish (PD-45, 50 mm in diameter, 11 mm in height; Advantec, Tokyo, Japan) containing 1.5 mL of a 0.25 M sucrose solution.

For the treatments with chemical mutagens, *N*-nitrosodimethylamine (NDMA), *N*-nitrosodiethylamine (NDEA), *N*-methyl-*N*-nitrosourea (MNU), *N*-ethyl-*N*-nitrosourea (ENU), and aflatoxin B_1_ were used. NDMA and NDEA were purchased from Tokyo Kasei Co., Ltd. (Tokyo, Japan), ENU and aflatoxin B_1_ were purchased from Sigma-Aldrich (St. Louis, MO, USA), and NMU was purchased from Nacalai Tesque (Kyoto, Japan). Third instar larvae were soaked in a 0.25 M sucrose solution containing NDMA or NDEA for 3 h, or MNU, ENU, or aflatoxin B_1_ for 6 h, as described previously [30].

The experiments on the exposure to tobacco smoke were performed according to our previous reports using an oxidative stress-sensitive *Drosophila* strain [31–33]. Approximately 500 third instar larvae in 0.25 M sucrose were placed in a plastic box with a nylon mesh cover, and were continuously exposed to tobacco smoke from three burning cigarettes for 6 h at 25 °C in an incubator (IJ300W, Yamato Scientific Co., Ltd., Tokyo, Japan).

UV-light irradiation was performed according to our previous reports [34, 35]. Four fluorescent-light lamps (FL 20SE, Toshiba, Tokyo, Japan) were used for the irradiation of polychromatic UVB light (wavelength: 300 to 400 nm, peak: 312 nm). The fluence (UV dose) was measured by a UVX-31 sensor attached to a UVX radiometer (UVP, Upland, CA, USA). Monochromatic UV-light irradiation (310, 320, 330, 340, and 360 nm) was carried out using the Okazaki Large Spectrograph at the National Institute for Basic Biology at Okazaki, Japan. The fluence was measured with a photon density meter, HK-1, that was manufactured by the Institute for Physical and Chemical Research (Wako, Saitama, Japan).

X-ray irradiation was performed according to our previous reports [36, 37] at non-toxic doses from 0 to 15 Gy using a Hitachi X-ray generator (MBR-1505R, Hitachi Medical Co., Tokyo, Japan) at 140 kV and 40 mA with a 1.0-mm Al filter at the Kindai University Atomic Energy Research Institute (Higashiosaka, Osaka, Japan).

### Wing-spot test

To detect somatic cell mutations, we performed the wing-spot test (somatic cell mutation and recombination test; SMART) established by Graf et al*.* [14] with slight modifications according to the methods described by Goto et al*.* [30]. In the wing-spot test, wild-type heterozygous larvae (*mwh/flr*) were prepared by mating virgin *mwh* females with *flr/TM1* males. The third instar larvae were collected at 72 to 96 h after oviposition. After treatment, larvae that were not to be used for the dissection of wing discs were reared on standard medium (Formula 4–24, Carolina Biological Supply, Burlington, NC, USA), and grown until the adult stage. The spots, which result from a hair shape that differs from the straight wild-type hairs, were scored on the wings of emerging adult flies. The wing-spot test that is linked to the DNA repair test was carried out according to our previous study using the *mei-9* or *mei-41* strain [35]. Statistical analysis was performed according to the methods of Frei and Würgler [38], and Kastenbaum and Bowman [39]. To examine whether mutated cells had proliferated, the distribution of spot sizes, which are the numbers of mutant cells in a spot, was analyzed according to the method of Graf et al*.* [14], and expressed as the average or mode spot size.

### Detection of apoptosis

#### DNA ladder assay

Twelve hours after treatment, approximately 50 wing discs were dissected from larvae in *Drosophila* Ringer’s solution (0.13 M NaCl, 5 mM KCl, and 2 mM CaCl_2_). The obtained wing discs were placed into lysis buffer (50 mM Tris–HCl (pH 7.5), 10 mM ethylenediaminetetraacetic acid, and 0.5% sodium-*N*-lauroyl sarcosinate), and incubated for 90 min at 50 °C. After treatment with RNase A for 30 min at 50 °C, then with proteinase K for 30 min at 50 °C, the lysate was obtained and used for electrophoresis on a 2% agarose gel containing ethidium bromide. The gel was analyzed by irradiation with a UV lamp.

#### TUNEL assay

The TUNEL method is widely used to detect apoptosis, as DNA fragmentation can be detected in situ by labeling the 3’-OH termini of DNA strand breaks using terminal deoxynucleotidyl transferase [40]. We detected DNA fragmentation in apoptotic cells in the wing discs, both in dissected wing discs and in 4-µm sections of paraffin-embedded larvae, by applying the modified TUNEL methods [41, 42] using the TdT-FragEL™ DNA Fragmentation Detection Kit (Oncogene Research Products, San Diego, CA, USA). Wing discs dissected in *Drosophila* Ringer’s solution were fixed with 10% formaldehyde in phosphate-buffered saline (PBS) for 5 min. After being washed with PBS containing 0.1% Triton-X, the wing discs were treated with 10 µg/mL Proteinase K (Merk KGaA, Darmstadt, Germany) for 10 min, then with H_2_O_2_ for 5 min. The discs were equilibrated in TdT Equilibration Buffer for 30 min, and the 3’ ends of the DNA fragments were labeled with biotin-bound deoxyribonucleotides for 3 h using terminal deoxynucleotidyl transferase. After peroxidase-streptavidin was conjugated to the labeled fragments, the wing discs were stained using a 3,3’-diaminobenzidine tetrachloride/Nickel–Cobalt Kit (Zymed Laboratories Inc., San Francisco, CA, USA). Black spots on wing discs were scored under a microscope (200 ×). Approximately 10 discs were scored for each sample.

Paraffin-embedded samples were prepared according to the methods of Fujikawa et al*.* [41]. The larvae were cut into two halves after treatment. The head parts of the larvae were fixed with 10% formaldehyde in *Drosophila* Ringer’s solution, and embedded in paraffin. Then, 4-µm sections were prepared from the paraffin-embedded block, mounted on slides, and stained using the Apoptaq® Peroxidase In situ Apoptosis Detection Kit (Sigma-Aldrich, Osaka, Japan) after removing the paraffin from the glass slides. The sections were counterstained with hematoxylin and eosin. Apoptotic cells were observed as dark brown spots. Stained sections were microscopically inspected at a magnification of 1000 × .

#### AO staining

As AO is excluded from viable cells [43], it has been used for the staining of Drosophila embryos and adult tissues to detect dead or dying cells [13, 43]. AO staining is easy and relatively fast to perform as fixation is not necessary [44]. We also applied AO to detect dead or dying cells in the wing discs. Dissected wing discs were soaked in 100 µL of 1 µM AO in Drosophila Ringer’s solution on a glass slide for a few minutes, then green fluorescent spots on the wing discs were scored under a fluorescence microscope at 200×. Approximately 10 discs were scored for each sample. When we examined apoptosis in two kinds of repair-deficient flies (*mei-9* and *mei-41*), only the wing discs of male larvae were selected by confirming the mouth hook color (dark brown) and gonadal shape, because only males are repair-deficient in these strains, as described in the “Drosophila strains” section of the Materials and Methods.

#### Detection of cell proliferation by labeling with 5-bromo-2’-deoxyuridine (BrdU)

Cell proliferation was detected by a BrdU-labeling method as described by Yamaguchi et al*.* [45, 46] with minor modifications. The wing discs dissected from third instar larvae were immediately incubated with 75 µg/mL BrdU in Grace’s insect medium (Invitrogen, Tokyo, Japan) for 30 min. These wing discs were fixed in Carnoy’s fixative (ethanol:acetic acid:chloroform = 6:1:3) for 15 min at 25 °C, and further fixed in 80% ethanol-50 mM glycine buffer (pH 2.0) at -20 °C overnight. The incorporated BrdU was detected using the BrdU Labeling and Detection Kit II (Roche Diagnostics, Basel, Switzerland). After treatment with anti-BrdU antibody at 4 °C overnight, BrdU was visualized by the reaction with anti-mouse IgG-alkaline phosphatase for 1 h and a color substrate solution (2.2 µL p-nitroblue tetrazolium chloride, 1.7 µL 5-bromo-4-chloro-3-indolyl phosphate, and 0.5 mL substrate buffer (100 mM Tris–HCl (pH 9.5), 100 mM NaCl, and 50 mM MgCl_2_)). These samples were mounted with 4% *n*-propyl gallate in 80% glycerol, and the level of BrdU incorporation was scored under a microscope at 200 × .

## Results

### Detection of DNA fragmentation by the DNA ladder assay

The detection of DNA ladders on agarose gels is a classical biochemical method to demonstrate apoptosis through nucleosomal fragmentation. We investigated whether DNA ladders could be observed in samples prepared from the larval wing discs at 12 h after irradiation with polychromatic UV light (UVB). DNA ladders with increments of 200 nucleotides were clearly observed when larvae were irradiated at 17.1 kJ/m^2^ and 34.2 kJ/m^2^; however, at these UVB doses, the rates of survival until adulthood were 10% and 0%, respectively (Supplemental Fig. 1). Therefore, it was concluded that this method for detecting apoptosis is not suitable for our aim, because we would not be able to accurately examine mutations at these toxic doses. Moreover, this method is not quantitative, and much time is needed to prepare the samples, as approximately 50 wing discs are necessary to detect DNA ladders.

### Detection of DNA fragmentation by the TUNEL assay

The TUNEL assay is widely used to detect apoptosis by DNA fragmentation. We applied this method on *Drosophila* larvae to detect DNA fragmentation in apoptotic cells in situ. First, we examined whether apoptotic cells could be detected in tissue sections prepared from paraffin-embedded larvae. When the larvae were irradiated with UV light (310-nm monochromatic UV light), we could detect spots stained by TUNEL on wing disc sections, but they were not detected on non-irradiated wing discs (Supplemental Fig. 2 (A)). As this method using paraffin-embedded sections of larvae is qualitative and not quantitative, we also applied the TUNEL assay on dissected wing discs. TUNEL-positive cells were observed as black clusters on the wing discs irradiated with X-ray, and they increased with the X-ray dose; in contrast, hardly any TUNEL-positive cells were observed on the non-irradiated wing discs (Supplemental Fig. 2 (B)). We scored these clusters to quantitatively determine the apoptotic level. The number of clusters on the discs from 10-Gy-irradiated larvae increased over time after irradiation, showing a peak at 12 h after irradiation, but no such change was seen in the non-irradiated discs (Supplemental Fig. 3 (A), Supplemental Table 1). UV-light (310-nm monochromatic UV) irradiation resulted in an apoptosis profile similar to that induced by X-ray irradiation (Supplemental Fig. 3 (B), Supplemental Table 2).

**Fig. 2:**
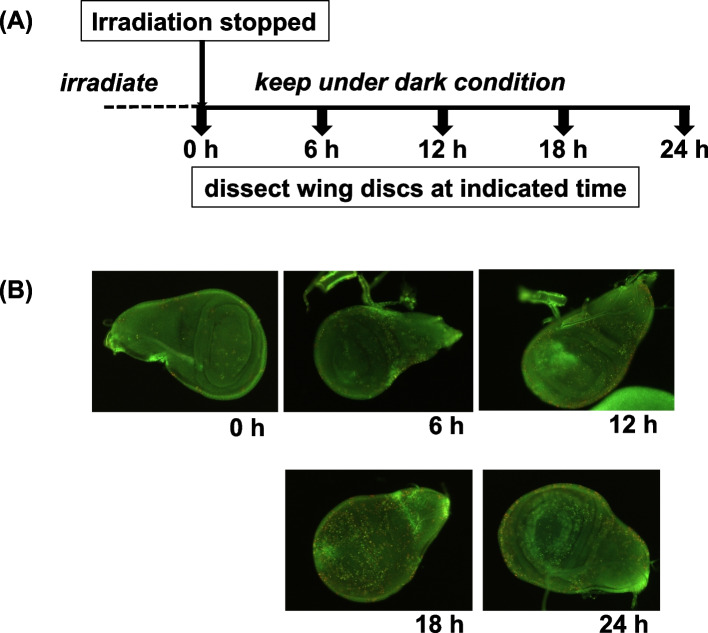
Detection of apoptosis by the AO-staining method. The wing discs were dissected from wild-type larvae (*mwh/flr*) that were irradiated with polychromatic UV light (peak at 312 nm) at 10 kJ/m^2^ according to the time schedule shown in (**A**). The dissected discs were soaked in *Drosophila* Ringer’s solution containing AO for a few minutes, then the fluorescent spots were observed (**B**). The indicated times are the time periods for which the larvae were kept in the dark after irradiation was stopped

**Fig. 3:**
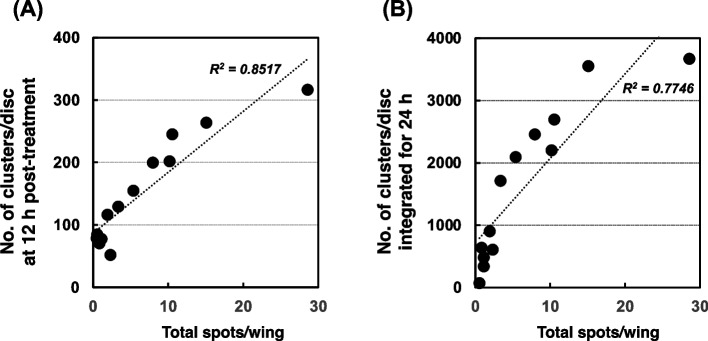
The relationship between mutagenicity and apoptosis in third instar larvae of wild-type (*mwh/flr*) treated with chemical mutagens as described in the Materials and Methods. The numbers of stained clusters were taken from Tables 1, 2, and 3. (**A**) The numbers of clusters per wing disc at 12 h post-treatment. (**B**) The total numbers of clusters per wing were integrated from the data from 0 to 24 h post-treatment. R^2^ is the correlation coefficient of the linear approximation. When the approximate curves were drawn from the scatter diagrams after the removal of the data for 10 mM MNU (toxic dose), the R^2^ was 0.9155 at 12 h post-treatment, and 0.904 for the 24-h integrated data

**Table 1:** Apoptosis and mutation induced by NDMA and NDEA for 3 h in wild-type *Drosophila* (*mwh/flr*)

	control	NDMA (µmol/dish)		NDEA (µmol/dish)	
	0	10	30	30	80
Apoptosis (number of clusters stained with AO/disc)					
Time post-treatment (h)					
0	14.8 ± 1.7	29.7 ± 17.1	42.9 ± 16.8	24.9 ± 11.1	41.1 ± 21.1
6	14.4 ± 7.1	79.1 ± 37.9	112.1 ± 52.2	45.9 ± 18.9	76.8 ± 26.8
12	21.0 ± 12.5	154.7 ± 70.0	244.8 ± 56.4	51.8 ± 9.6	129.0 ± 35.4
18	15.6 ± 8.4	188.9 ± 61.7	170.9 ± 61.5	71.9 ± 36.0	146.4 ± 46.1
24	23.5 ± 10.1	60.6 ± 27.4	141.9 ± 40.4	38.1 ± 9.0	153.4 ± 36.6
Mutation (number of total spots/wing)					
	0.53	5.39**	10.6**	2.13**	3.35**
Survival (%)					
	100	134	94.6	92.2	52.7

^**^P < 0.01, a significant difference from the corresponding control (without treatment)

**Table 2:** Apoptosis and mutation induced by MNU and ENU for 6 h in wild-type *Drosophila* (*mwh/flr*)

	control	MNU (µmol/dish)		ENU (µmol/dish)	
	0	3	10	10	30
Apoptosis (number of clusters stained with AO/disc)					
Time post-treatment (h)					
0	28.6 ± 11.0	74.6 ± 30.5	95.7 ± 19.1	54.6 ± 20.7	60.8 ± 22.7
6	54.8 ± 18.9	223.1 ± 74.2	274.8 ± 110.6	180.2 ± 58.6	289.5 ± 74.3
12	78.7 ± 26.7	199.7 ± 66.6	316.1 ± 85.4	201.9 ± 66.1	263.7 ± 86.5
18	35.2 ± 12.8	118.9 ± 80.9	256.7 ± 91.1	117.4 ± 42.4	162.3 ± 46.7
24	41.3 ± 19.0	116.8 ± 46.6	196.7 ± 54.2	121.4 ± 39.2	160.2 ± 67.7
Mutation (number of total spots/wing)					
	0.49	7.96**	28.6**	10.2**	15.1**
Survival (%)					
	100	100	44.9	83.2	77.1

^**^P < 0.01, a significant difference from the corresponding control (without treatment)

### Detection of apoptosis by AO staining

For the detection of apoptotic cells, AO staining is easier to perform than the TUNEL assay since the wing discs do not have to be fixed. Results can be obtained quickly (within only a few minutes) by staining the wing discs on a glass slide, making it a more convenient method. Clusters of apoptotic cells were seen as spots of green fluorescence (Fig. 2). After the larvae were irradiated with polychromatic UV light (peak at 312 nm), hardly any fluorescent spots were observed just after irradiation was stopped (0 h), but they appeared and increased gradually over time as the larvae were incubated in the dark. In wild-type *Drosophila*, the fluorescent spots could be counted until 24 h after irradiation, and the number of spots reflected the intensity of apoptosis; following irradiation, the number of fluorescent spots increased, peaked at 12 h, and subsequently decreased. A similar profile of AO-stained spots was also observed with 310-nm monochromatic UV-light irradiation as well as with X-ray irradiation. In the controls (0 h, 0 Gy, or 0 J), the number of clusters of AO-stained apoptotic cells was low when compared to that of the apoptotic cell clusters detected by the TUNEL assay. The profile of apoptosis was similar to that observed in the TUNEL assay, *i.e.,* the number of apoptotic cells increased at 6 to 12 h, then decreased. From the results comparing the AO-staining method to the TUNEL assay, the AO-staining method appeared to be more sensitive for detecting apoptosis than the TUNEL assay (Supplemental Fig. 3). AO staining was found to be the easiest, quickest, and most sensitive method for determining in situ the profiles of apoptosis induced by treatments with mutagenic factors. Therefore, in this study, we adopted the AO-staining method of detecting apoptosis to investigate the relationship between mutation and apoptosis.

### Apoptosis and mutation induced by chemical mutagens

When we previously examined the mutagenicity of *N*-nitroso compounds using the wing-spot test, methylating agents showed a much stronger mutagenic effect than ethylating agents [30]. When we examined apoptosis in wing discs by scoring the AO-stained clusters, a remarkably stronger apoptotic effect was seen with NDMA than NDEA (Table 1). Similar results were obtained from the comparison between MNU and ENU, *i.e.,* MNU showed a stronger apoptotic effect than ENU (Table 2). Aflatoxin B_1_ is a strong mutagen in the Ames test; however, it was not so mutagenic in *Drosophila*, and weakly induced apoptosis (Table 3). In general, the number of apoptotic clusters increased, peaked at 12 h after the treatment was stopped, and decreased gradually thereafter, except for when a strong toxic effect was observed. When the average number of AO-stained clusters at 12 h post-treatment was plotted against the total number of spots per wing from the data in Tables 1, 2, and 3, we found that the apoptosis induction potency and mutagenicity were correlated in a linear relationship, as shown in Fig. 3 (A), although apoptosis appeared to become saturated at the highest mutagenicity (MNU at 10 mM). When the numbers of AO-stained clusters were combined from 0 to 24 h as the total number of apoptotic clusters induced during 24 h after treatment, then plotted against the mutagenicity, the relationship between apoptosis and mutation was similar to that determined using the data of apoptotic clusters at 12 h post-treatment (Fig. 3 (B)) or 6 h post-treatment (data not shown). Therefore, we decided to use the number of AO-stained clusters obtained at 12 h post-treatment as the value of the apoptosis induction potency.

**Table 3:** Apoptosis and mutation induced by aflatoxin B_1_ for 6 h in wild-type *Drosophila* (*mwh/flr*)

	Aflatoxin B_1_ (nmol/dish)			
	0	50	100	500
Apoptosis (number of clusters stained with AO/disc)				
Time after the treatment was stopped (h)				
0	28.6 ± 11.0	56.5 ± 25.3	52.6 ± 22.0	87.2 ± 27.5
6	54.8 ± 18.9	60.3 ± 28.3	83.3 ± 34.1	94.4 ± 34.6
12	78.7 ± 26.7	70.2 ± 15.6	77.3 ± 19.5	116.2 ± 29.7
18	35.2 ± 12.8	46.8 ± 10.6	53.2 ± 12.9	103.0 ± 41.1
24	41.3 ± 19.0	28.4 ± 10.3	53.8 ± 22.7	59.1 ± 27.5
Mutation (number of total spots/wing)				
	0.49	0.84*	1.14*	1.92*
Survival (%)				
	100	101	102	99.1

^*^P < 0.05, a significant difference from the control (without treatment)

We also examined whether exposure to cigarette smoke, *i.e.,* a mixture of chemical mutagens and carcinogens, induces apoptosis in *Drosophila* larvae. We reported previously that exposure to cigarette smoke induced mutation in an oxidative damage-sensitive strain (*y v ma-l*), but not in a wild-type strain (Oregon-R) [33]. Apoptosis was weakly, but significantly (*P* < 0.005) induced even at 6 h post-exposure in the oxidative damage-sensitive strain, but not in the wild-type strain (Table 4).

**Table 4:** Apoptosis and mutation induced by exposure to cigarette smoke (CS) for 6 h in wild-type *Drosophila* (Oregon-R) and oxidative damage-sensitive strain *Drosophila* (*y v ma-l*)

Strain	Oregon-R		*y v ma-l*	
Time of exposure to CS	0	6 h	0	6 h
Apoptosis (number of clusters stained with AO/disc) ^*a*^				
Time post-exposure (h)				
0	26.9 ± 11.3	24.1 ± 12.5	30.6 ± 10.9	19.0 ± 8.2
6	22.7 ± 9.6	27.7 ± 21.6	26.3 ± 13.8	41.3 ± 29.6
12	19.9 ± 5.8	23.2 ± 13.7	22.1 ± 9.7	41.4 ± 19.9
18	21.3 ± 8.7	26.9 ± 11.0	19.1 ± 8.2	32.4 ± 14.6
24	18.4 ± 9.5	24.0 ± 13.2	21.5 ± 9.2	24.8 ± 9.4

*a*: The number of discs scored was 30. Ten discs were counted for each experiment, and three independent experiments were performed

### Apoptosis and mutation induced by UV irradiation

Previously, we reported on the somatic cell mutations induced by UV irradiation using the *Drosophila* wing-spot test [34, 35]. We used polychromatic UVB (peak at 312 nm) and monochromatic UV light (310, 320, 340, and 360 nm) as UV light sources. The *Drosophila* strains used included two wild-type strains (*mwh/flr* and *mei*^+^), three excision repair-deficient strains (*mus201, mei-9* and *mus201; mwh/flr***)**, and a post-replication repair-deficient strain (*mei-41*).

**Fig. 4:**
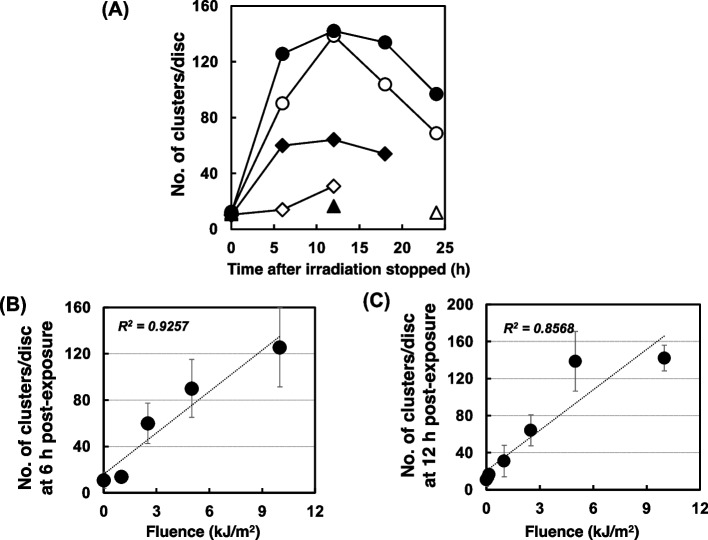
Apoptosis on wild-type (*mwh/flr*) wing discs induced by irradiation with UVB using four fluorescent lamps (peak at 312 nm). (**A**) The number of AO-stained clusters induced by irradiation at 0 kJ/m^2^ (open triangles), 0.15 kJ/m^2^ (closed triangles), 1 kJ/m^2^ (open diamonds), 2.5 kJ/m^2^ (closed diamonds), 5 kJ/m^2^ (open circles), and 10 kJ/m^2^ (closed circles). (**B**) and (**C**) The UV-dose dependency of apoptosis at 6 h post-exposure (**B**) and 12 h post-exposure (**C**). Apoptosis is shown as the number of clusters stained by AO. The values of R^2^ were obtained by a linear approximation method

**Fig. 5:**
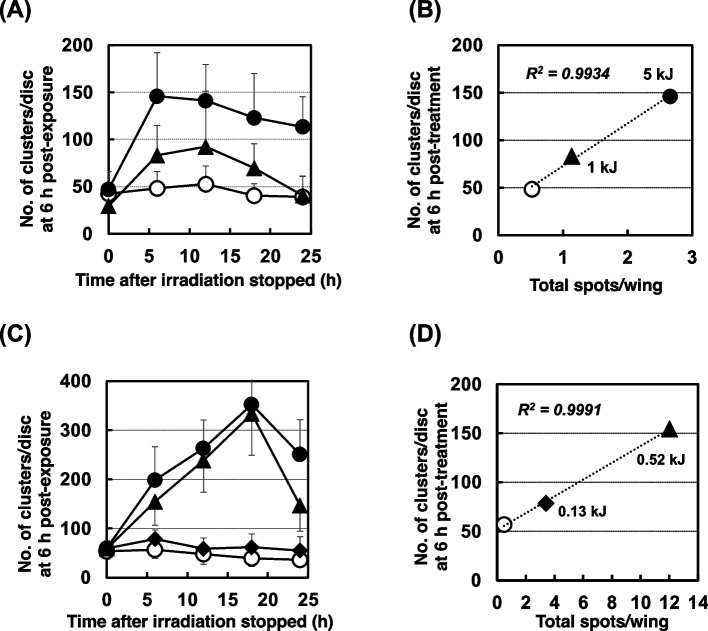
Effects of polychromatic UVB irradiation on the wild-type and NER-deficient *Drosophila*. (**A**) The number of AO-stained clusters induced by UVB-irradiation at 0 kJ/m^2^ (open triangles), 1 kJ/m^2^ (closed triangles), and 5 kJ/m^2^ (closed circles) in wild-type larvae (*mwh/flr*). (**B**) The relationship between the number of AO-stained clusters per wing and total spots per wing at each UV dose in the wild-type flies (symbols are the same as in panel (A)). (**C**) The number of AO-stained clusters induced by UVB-irradiation at 0 kJ/m^2^ (open triangles), 0.13 kJ/m^2^ (closed diamonds), 0.52 kJ/m^2^ (closed triangles), and 1.3 kJ/m^2^ (closed circles) in NER-deficient larvae (*mus201; mwh/flr*). (**D**) The relationship between the number of AO-stained clusters per wing and total spots per wing at each UV dose in the NER-deficient flies (*mus201; mwh/flr*) (symbols are the same as in panel (C))

Figure 4 shows the profile of apoptosis after polychromatic UVB irradiation in the wild-type *Drosophila*. The number of AO-stained clusters increased dose-dependently, peaking at 12 h, then decreasing at every dose from 0 to 10 kJ/m^2^ (Fig. 4 (A)). The dose-dependency of the AO-stained clusters both at 6 h and 12 h post-irradiation is shown in Fig. 4 (B) and (C), respectively. The number of AO-stained clusters appeared to become saturated at high doses. When the data of the 10 kJ/m^2^ condition were removed, the relationship between apoptosis and the UV dose showed a more linear equation at 6 h (R^2^ = 0.9483) and 12 h (R^2^ = 0.9914). When mutation was observed in the same lot of wild-type flies in which apoptosis was observed after the polychromatic UVB irradiation, as shown in Fig. 5 (A) and (B), we found that the apoptosis induction potency and mutagenicity were well-correlated in a linear relationship (R^2^ = 0.9934), similar to the case of chemical mutagens.

**Fig. 6:**
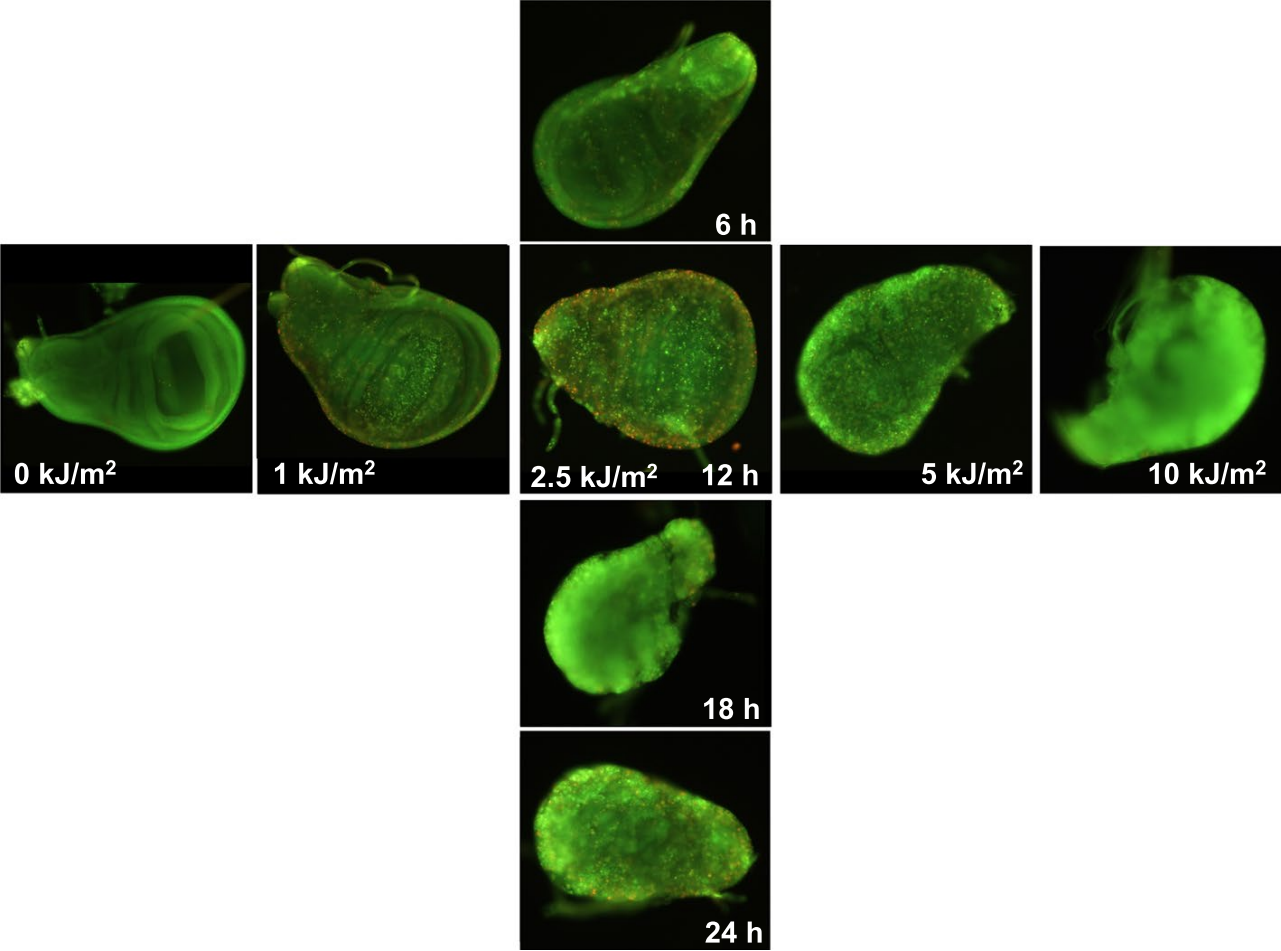
Photos of wing discs stained with AO that were dissected from the NER-deficient larvae *(mus201*). The NER-deficient larvae *(mus201*) were irradiated with polychromatic UVB. The horizontal line shows the wing discs from larvae 12 h after irradiation at 0 to 10 kJ/m^2^. The vertical line shows the wing discs from larvae kept for the indicated times after irradiation at 2.5 kJ/m^2^

To observe the effect of DNA repair deficiency on the induction of apoptosis and mutation due to UV light irradiation, we performed experiments using a NER-deficient *Drosophila* strain (*mus201* and *mus201; mwh/flr* for the wing spot test). When *mus201* larvae were irradiated with polychromatic UVB light, AO-stained fluorescent spots were observed on wing discs (Fig. 6). Apoptotic clusters immediately and continuously increased dose-dependently, and the discs lost their shapes at low UV doses that were non-toxic for wild-type *Drosophila*. The number of apoptotic clusters increased time-dependently after treatment at 1 and 2.5 kJ/m^2^ (Supplemental Fig. 4 (A)). At 1 kJ/m^2^, the number of AO-stained clusters increased continuously, and unlike in the wild-type *Drosophila*, it did not decrease thereafter; at 2.5 kJ/m^2^, the discs lost their shapes after 12 h. As shown in Fig. 5 (C) and (D), we also found that the apoptosis induction potency and mutagenicity were well-correlated in a linear relationship (R^2^ = 0.9991) even in the NER-deficient strain at non-toxic doses. At the highest dose (1.3 kJ/m^2^) in Fig. 5 (C), there were hardly any AO-stained clusters to score due to the toxicity, and we could not score mutant spots due to the lethality (Supplemental Fig. 4 (C)).

**Fig. 7:**
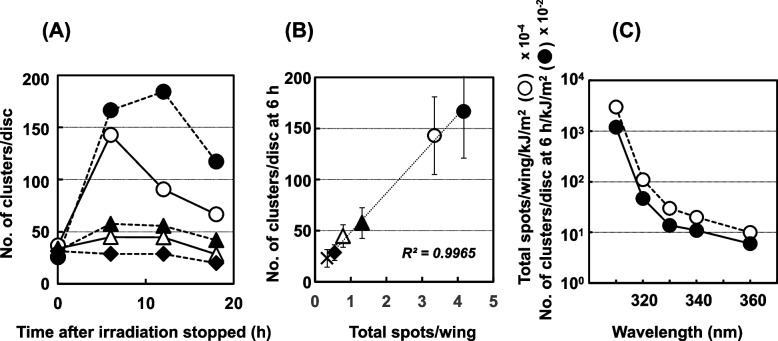
Apoptosis and mutation induced by monochromatic UV light in wild-type *Drosophila* (*mwh/flr*). Apoptosis was determined by the number of clusters stained with AO. (**A**) The profiles of apoptosis induced by monochromatic UV irradiation with 310-nm light at 14 kJ/m^2^ (closed circles), 320-nm light at 305 kJ/m^2^ (open circles), 330-nm light at 419 kJ/m^2^ (closed triangles), 340-nm light at 406 kJ/m^2^ (open triangles), 360-nm light at 477 kJ/m^2^ (closed diamonds), or no irradiation (cross). (**B**) The relationship between the number of AO-stained clusters per wing and total spots per wing at each wavelength (symbols are the same as in panel (A)). The values of R^2^ were obtained by a linear approximation method. (**C**) The action spectra of the mutagenicity were determined as the total spots per wing normalized by the UV fluence (open circles) as described previously [47], and apoptosis inducibility was determined as the number of clusters per wing disc at 6 h normalized by the UV fluence (closed circle)

Monochromatic UV light also resulted in a profile of apoptosis similar to that of polychromatic UV light at every wavelength tested, except for 360 nm, in the wild-type *Drosophila* (Table 5). Interestingly, the apoptotic events appeared to occur just after irradiation was stopped, independently of the irradiation period, at each wavelength tested (30 min with 310-nm light, or 6 h with other wavelengths of light). The intensity of the apoptotic effect of the 330- and 340-nm light was lower than that of the 310- and 320-nm light, although the fluence of the 330- and 340-nm light was higher than that of the 310- and 320-nm light (Fig. 7 (A)). Simultaneously, we scored the mutagenicity of each wavelength (Table 5). The mutagenicity of the 330- and 340-nm light was significantly lower than that of the 310- and 320-nm light, and the 360-nm light showed no mutagenicity. The number of clusters per disc (apoptosis) was extremely well-correlated with the total spots per wing (mutagenicity) in a linear equation (Fig. 7 (B)), but it was not correlated with the UV fluence, *i.e.,* the energy of the 310-nm light was much smaller than that of the light with a wavelength of 320 nm and over. When the apoptosis induction potency and mutagenicity were normalized by the UV fluence, their action spectra were parallel (Fig. 7 (C)).

**Table 5:** Apoptosis and mutation induced by monochromatic UV light in wild-type *Drosophila* (*mwh/flr*)

	Wavelength (nm)					
	Control	310	320	330	340	360
Fluence (kJ/m^2^)	0	14	305	419	406	477
Apoptosis (number of clusters stained with AO/disc)						
Time post-exposure (h)						
0	22.1 ± 8.6	25.3 ± 7.4	36.7 ± 17.8	31.7 ± 11.5	33.7 ± 22.3	31.3 ± 7.8
6	-	166.6 ± 45.8	142.9 ± 38.0	57.5 ± 15.0	44.8 ± 11.0	28.6 ± 7.7
12	-	184.3 ± 24.4	90.5 ± 24.3	55.5 ± 10.9	44.5 ± 12.8	28.7 ± 8.5
18	23.5 ± 8.3	117.3 ± 46.3	66.7 ± 17.8	41.8 ± 10.2	28.3 ± 6.9	19.8 ± 5.1
Mutation (number of total spots/wing)						
	0.34	4.17**	3.35**	1.32**	0.79**	0.55
The number of AO clusters at 6 h/wing/kJ/m^2^ × 10^–2^						
	-	11.9	0.468	0.137	0.110	0.060
Total spots/wing/kJ/m^2^ × 10^–2^						
	-	29.8	1.10	0.315	0.195	0.115

^**^P < 0.01, a significant difference from the control (without irradiation)

**Fig. 8:**
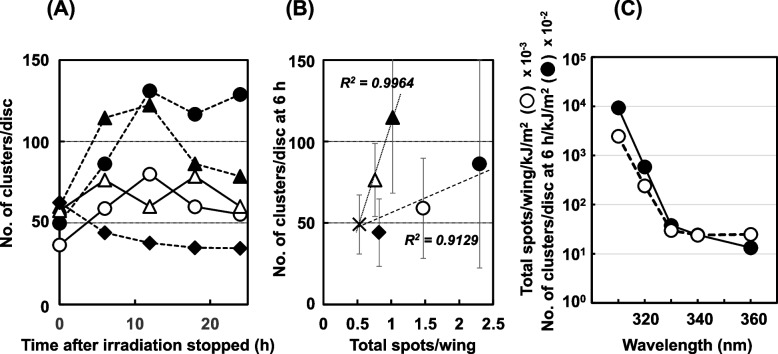
Apoptosis and mutation induced by monochromatic UV light in NER-deficient *Drosophila* (*mus201; mwh/flr*). (**A**) The profiles of apoptosis induced by UV irradiation with 310-nm light at 0.93 kJ/m^2^ (closed circles), 320-nm light at 10 kJ/m^2^ (open circles), 330-nm light at 302 kJ/m^2^ (closed triangles), 340-nm light at 315 kJ/m^2^ (open triangles), 360-nm light at 330 kJ/m^2^ (closed diamonds), or no irradiation (cross). (**B**) The relationship between the number of AO-stained clusters per wing and total spots per wing at each wavelength (symbols are the same as in panel (A)). (**C**) The action spectra of the mutagenicity determined as the total spots per wing normalized by the UV fluence (kJ/m^2^), and apoptosis inducibility (open circles), which was determined as the number of clusters per wing disc at 6 h normalized by the UV fluence (kJ/m^2^; closed circles)

When we used the NER-deficient strain *mus201; mwh/flr*, the profile of apoptosis and the relationship with mutation differed from those of the wild-type *Drosophila* (Table 6 and Fig. 8). The number of AO-stained clusters increased continuously over 24 h post-irradiation with 310-nm light, and the shape of the discs collapsed in a post-irradiation time-dependent manner. With the irradiation of 320- and 330-nm light, apoptosis increased, then decreased; in contrast, no obvious apoptosis was detected with the irradiation of 360-nm light. No correlation was seen between the apoptosis induction potency and mutagenicity at every wavelength tested, unlike in the wild-type *Drosophila* (Fig. 8 (B)). The action spectrum of the apoptosis induction potency was not parallel to that of mutagenicity, unlike in the wild-type *Drosophila* (Fig. 8 (C)). When we used another NER-deficient strain, *mei-9*, the profile of apoptosis induced by UV differed from that in *mus201*. In *mei-9*, apoptosis increased in a post-irradiation time-dependent manner at every wavelength tested (310 to 360 nm), except in the non-irradiated samples (Supplemental Fig. 5 (A)). In contrast, in the post-replication repair-deficient strain *mei-41*, apoptosis increased, then decreased in a similar manner as in the wild-type *Drosophila* (Supplemental Fig. 5 (B)), differently from the profile of apoptosis at polychromatic UVB irradiation, where AO-staining clusters increased continuously (Supplemental Fig. 4 (B)).

**Table 6:** Apoptosis and mutation induced by monochromatic UV light in NER-deficient *Drosophila* (*mus201; mwn/flr*)

	Wavelength (nm)					
	Control	310	320	330	340	360
Fluence (kJ/m^2^)	0	0.93	10	302	315	330
Apoptosis (number of clusters stained with AO/disc)						
Time post-exposure (h)						
0	-	49.9 ± 15.4	36.5 ± 9.7	60.2 ± 32.4	57.6 ± 17.2	62.6 ± 25.4
6	49.1 ± 18.1	86.3 ± 63.9	59.0 ± 30.8	114.6 ± 46.3	76.4 ± 22.4	44.1 ± 20.7
12	54.6 ± 7.9	131.2 ± 61.6	80.0 ± 34.5	122.5 ± 54.4	60.2 ± 21.9	37.8 ± 18.0
18	29.4 ± 6.4	116.8 ± 83.3	60.0 ± 30.7	86.3 ± 46.2	78.7 ± 32.1	34.8 ± 12.1
24	25.5 ± 8.6	129.0 ± 46.0	55.4 ± 31.8	78.6 ± 23.5	60.2 ± 24.9	34.5 ± 17.2
Mutation (number of total spots/wing)						
	0.53	2.30**	1.47**	1.02**	0.76**	0.82*
The number of AO clusters at 6 h/wing/kJ/m^2^						
	-	92.8	5.90	0.379	0.243	0.134
Total spots/wing/J/m^2^						
	-	2.473	0.147	0.00338	0.00241	0.00248

^*^P < 0.05, **P < 0.01, a significant difference from the control

### Apoptosis and mutation induced by X-ray irradiation

**Fig. 9:**
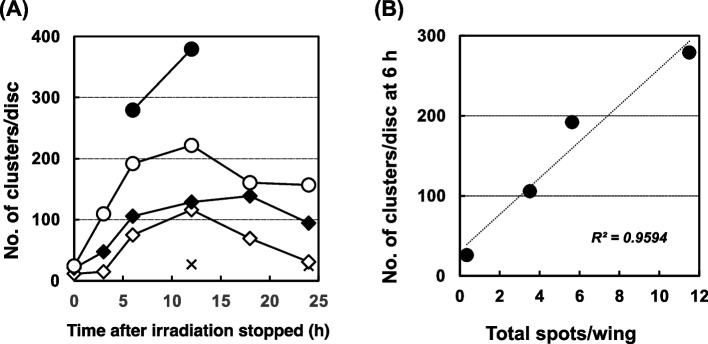
Effects of X-ray irradiation on wild-type *Drosophila* (*mwh/flr*). (**A**) Appearance of AO-stained clusters on wing imaginal discs after radiation at 2.5 Gy (open diamonds), 5 Gy (closed diamonds), 10 Gy (open circles), and 15 Gy (closed circles). Crosses show the data of the non-irradiated samples. (**B**) The relationship between the number of AO-stained clusters per wing and total spots per wing. The values of R^2^ were obtained by a linear approximation method

To investigate whether X-ray irradiation induces mutation and/or apoptosis at non-toxic doses, we examined the effects of 0 to 15 Gy of irradiation on wild-type flies. Apoptosis was detectable even at 3 h after irradiation, and it increased until 12 h, then decreased. With 2.5 Gy of irradiation, apoptosis decreased to a level similar to that observed in non-irradiated larvae at 24 h (Fig. 9 (A)). There was a correlation between the number of apoptotic clusters at 12 h and the number of mutant spots, similar to other mutagenic factors (Fig. 9 (B); R^2^ = 0.9594). When we used NER-deficient strain, *mus201*, apoptosis increased and decreased in the similar manner to wild-type (Supplemental Fig. 6 (A)). In contrast, in the post-replication repair-deficient strain *mei-41*, apoptosis increased continuously (Supplemental Fig. 6 (B)). These profiles of apoptosis reflected the toxicity (Supplemental Fig. 6 (C)).

### Distribution of spot sizes after treatment with MNU, UV, and X-ray

**Fig. 10:**
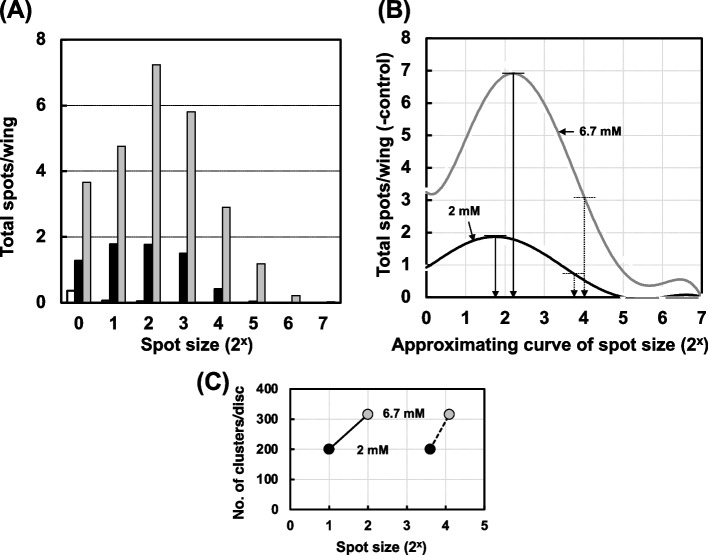
(**A**) Distribution of mutant spot sizes observed on the wings of larvae (*mwh/flr*) treated with MNU at 2 mM (black bars), 6.7 mM (gray bars), and 0 mM (white bars). (**B**) Approximation curves of the spot size fitted by the polynomial approximation for the larvae treated with MNU at 2 mM (black line) and 6.7 mM (gray line). The mode of the spot size (solid arrows; 1.8 for 2 mM MNU and 2.2 for 6.7 mM MNU) and the average of the spot size (dotted arrows; 3.6 for 2 mM MNU and 4.1 for 6.7 mM MNU) for each dose were calculated from each polynomial approximation curve after the subtraction of the spot number without irradiation. (**C**) The relationship between the number of AO-stained clusters per wing and the mode of the spot size (solid lines) or the average of the spot size (dotted lines). The concentrations of MNU were 2 mM (black circles) and 6.7 mM (gray circles)

**Fig. 11:**
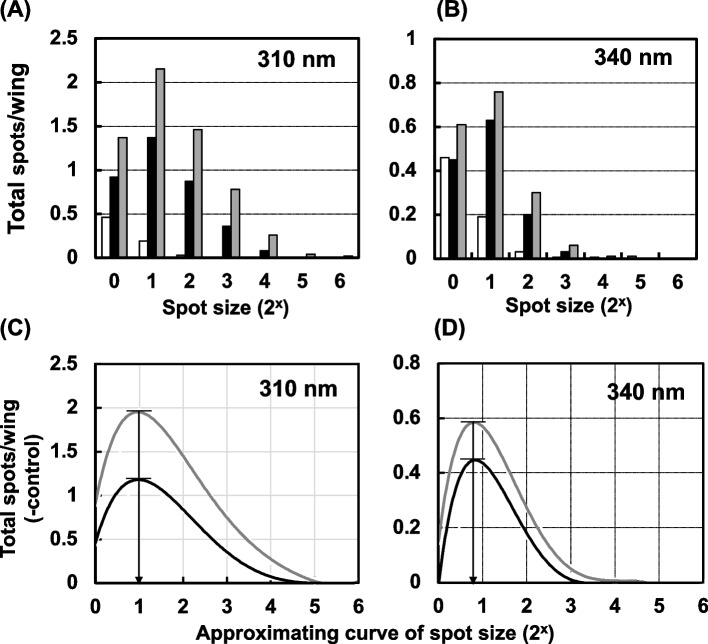
Distribution of mutant spot sizes on the wings from larvae irradiated with monochromatic UV light. (**A**) Wild-type larvae (*mwh/flr*) were irradiated with 310-nm light at 12.9 kJ/m^2^ (black bars) and 25.7 kJ/m^2^ (gray bars), or no irradiation (white bars). (**B**) Wild-type larvae (*mwh/flr*) were irradiated with 340-nm light at 267 kJ/m^2^ (black bars), 531 kJ/m^2^ (gray bars), or no irradiation (white bars). (**C**) Approximation curves of the spot size fitted by the polynomial approximation for 310-nm light at 12.9 kJ/m^2^ (black line) and 25.7 kJ/m^2^ (gray line). The mode of the spot size (1.0) was calculated from each polynomial approximation curve after the subtraction of the spot number without irradiation. (**D**) Approximation curves of the spot size fitted by the polynomial approximation for the irradiation with 340-nm light at 267 kJ/m^2^ (black line) and 531 kJ/m^2^ (gray line). The mode of the spot size (0.8) was calculated from each polynomial approximation curve after the subtraction of the spot number without irradiation

In the wing-spot test, the number of mutant cells contained in a spot, which is referred to as the spot size, indicates the frequency of cell division [14]. To investigate the relationship between apoptosis and mutated cell proliferation, we analyzed the spot size, and calculated the average and mode of the spot size from the approximation curve of spot size fitted by the polynomial approximation at each dose of the mutagens after treatment with MNU, UV, or X-ray. As shown in Fig. 10, the spot size became larger with increasing MNU dose; the average of the spot size was estimated to be 3.6 at 2 mM, and 4.1 at 6.7 mM, and the mode of the spot size was 1.8 at 2 mM, and 2.2 at 6.7 mM (Fig. 10 (B)). These spot sizes were well-correlated with the number of apoptotic clusters (Fig. 10 (C)). In contrast, with UV irradiation, the spot size distribution remained fairly constant regardless of the experimental conditions, *i.e.,* it was unrelated to the UV dose and wavelength (Fig. 11 (A) and (B)). The mode of the spot size estimated from the approximation curve was the same (1 at 310 nm, and 0.8 at 340 nm) at the two UV doses of each wavelength (Fig. 11 (C) and (D)). Even in the different kinds of repair-deficient *Drosophila* strains, the spot size did not change at every UV wavelength tested (Supplemental Fig. 7 (A) and (B)).

**Fig. 12:**
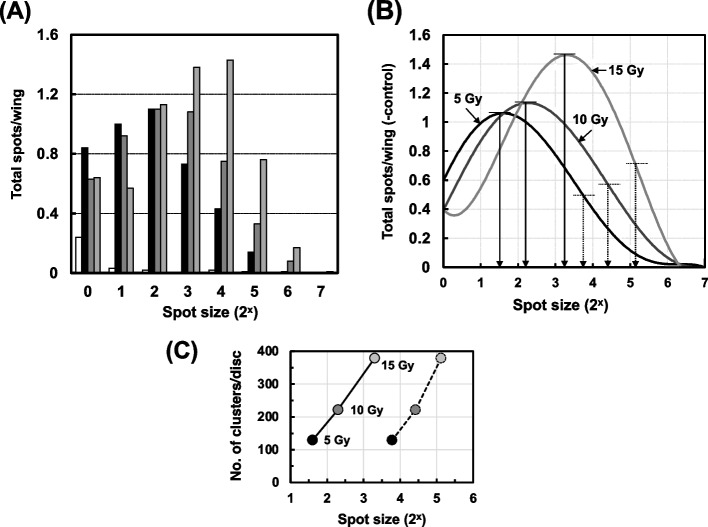
(**A**) Distribution of mutant spot sizes observed on the wings from the experiments described in Fig. 9. The numbers of total spots shown are from larvae irradiated at 15 Gy (gray bars), 10 Gy (dark bars), 5 Gy (black bars), or 0 Gy (no irradiation; white bars). (**B**) Approximation curves of the spot size fitted by the polynomial approximation for the irradiation at 5 Gy (black line), 10 Gy (dark line), and 15 Gy (gray line). The mode of the spot size (solid arrows; 1.6 for 5 Gy, 2.3 for 10 Gy, and 3.3 for 15 Gy) and the average of the spot size (dotted arrows; 3.8 for 5 Gy, 4.4 for 10 Gy, and 5.1 for 15 Gy) were calculated from each polynomial approximation curve after the subtraction of the spot number without irradiation. (**C**) The relationship between the number of AO-stained clusters per wing and the mode of the spot size (solid lines) or average of the spot size (dotted lines). The X-ray doses were 5 Gy (black circles), 10 Gy (dark circles), and 15 Gy (gray circles)

In the case of X-ray irradiation (Fig. 12 (A)), the spot size became markedly larger when the X-ray dose was higher, implying that higher doses of irradiation induced higher levels of proliferation. When we estimated the average and mode of the spot size from the approximation curve of the spot size fitted by the polynomial approximation at each X-ray dose (Fig. 12 (B)), both the average and mode of the spot size showed a good correlation with the number of clusters stained with AO, as shown in Fig. 12 (C). From these results, we consider that the mutated cells induced by MNU or X-ray irradiation could proliferate in parallel with the increase in apoptotic cell death, but that the mutated cells induced by UV irradiation could not.

**Fig. 13:**
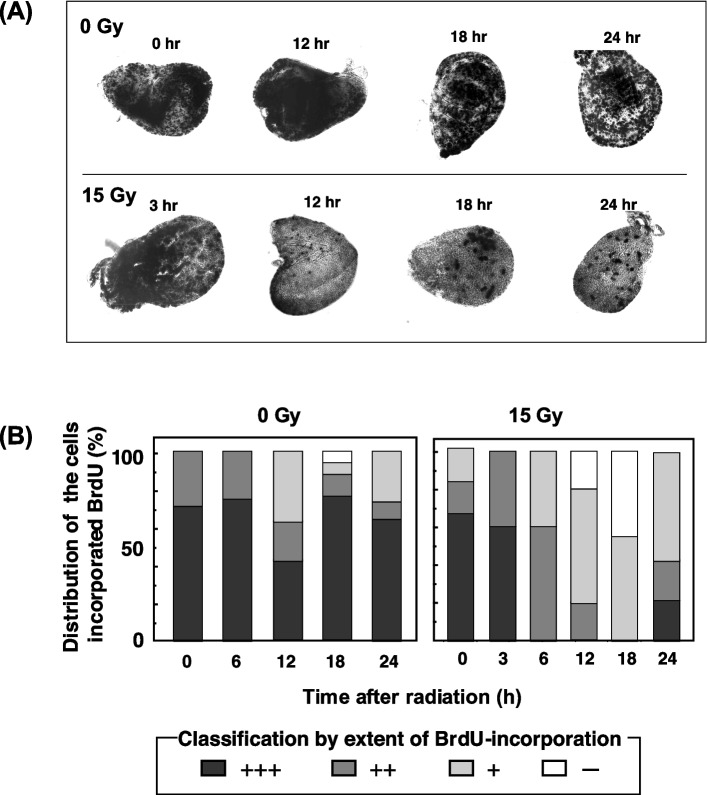
Analysis of cell proliferation. (**A**) BrdU-incorporated wing discs after X-ray irradiation in wild-type *Drosophila* (*mwh/flr*). Black spots show the areas with incorporated BrdU. Discs appear typical at each time point in the absence of irradiation (0 Gy) and post-irradiation (15 Gy). (**B**) The level of BrdU incorporation after irradiation. Wing discs were grouped according to the level of BrdU incorporation: +  +  + indicates the level of the non-irradiated stained disc shown in (A); +  + , + , and—indicate the discs at 3, 24, and 12 h after 15-Gy irradiation, respectively, shown in (**A**). Approximately 20 discs were observed and classified into four groups for each time point

To explore how these mutagens affect cell proliferation in the wing discs of third instar larvae, we measured the incorporation of BrdU in the larval wing discs after X-ray irradiation. Because larval disc cells proliferate efficiently, wing discs from larvae without radiation incorporated a significant amount of BrdU over a 24-h period (Fig. 13 (A). In contrast, the incorporation of BrdU in discs from larvae irradiated with 15 Gy appeared to vary with time after irradiation. At 6 h post-irradiation, when apoptosis was readily detectable, the proportion of discs with active BrdU incorporation decreased, and it continued to decrease until 12 to 18 h post-irradiation, suggesting that cell proliferation had stopped in these discs. However, at 18 to 24 h post-irradiation, we observed clusters of cells that were positive for BrdU in the discs (Fig. 13 (A)), suggesting that at least some cells in the discs had started to proliferate again. The distribution of wing discs according to the level of BrdU incorporation is shown in Fig. 13 (B). When larvae were irradiated with UV, the BrdU-incorporation level did not obviously change at the different time points after irradiation (data not shown).

## Discussion

It is well-documented that apoptosis, as a programmed cell death process, plays important roles in the development of multicellular organisms, including *Drosophila* [13]. However, the relationship between apoptosis and mutation following DNA damage, and the involvement of DNA repair in apoptosis remain unclear. It is important to investigate the relationship between apoptosis and mutation to gain a better understanding of the defense system against DNA damage. In this study, we investigated whether the occurrence of apoptosis is involved in the induction of somatic cell mutations due to various mutagenic factors, such as chemical mutagens, UV light, and X-ray, in *Drosophila*. In addition, we also examined how apoptotic events are correlated with cell proliferation.

In the wild-type *Drosophila* larvae treated at non-toxic doses of mutagens, apoptosis began to increase just after treatment was stopped (0 h), peaked at 6 to 12 h, then decreased to levels close to those of the non-treated controls at 24 h. In *Drosophila*, as cell division occurs only at the embryo, imaginal disc cells in larva and stem cells of adult fly, it is considered that mutagenic reactions against DNA damage should have been completed by 24 h in the third instar larvae used in this study. The apoptosis-induction level showed a good correlation with the doses of the mutagenic factors. As was expected, a good relationship was found between apoptosis and somatic cell mutation, determined as the total spots/wing, at non-toxic doses of the mutagens. In the experiment with monochromatic UV light, the number of AO-stained clusters observed on wild-type larval wing discs showed a linear correlation with the number of total spots, regardless of the wavelength (Fig. 7 (B)), and the action spectra of apoptosis and mutation were parallel (Fig. 7 (C)). These data suggest that the reaction against DNA damage, leading to mutation or apoptosis, occurs in a similar manner at every wavelength. Even in the NER-deficient *Drosophila*, the good correlation between apoptosis and mutation was observed (Fig. 5 (D)), although the slope of the approximate line became less steep when compared to that of the wild-type *Drosophila*. These results indicate that mutagenic events were more frequent in the NER-deficient *Drosophila* than in the wild-type *Drosophila* at the same level of apoptosis, suggesting that the balance between the apoptosis induction potency and mutagenicity varies depending on the repair system of DNA damage. When larvae could not repair DNA damage due to the lack of repair system, larvae might intend to be mutated to survive. When we observed the induction of apoptosis due to irradiation with monochromatic UV light from 310 to 360 nm in the NER-deficient *Drosophila*, the profiles of apoptosis differed from those in wild-type *Drosophila*; in particular, at 310 nm, apoptosis increased continuously. When we separated the wavelengths into shorter (310 and 320 nm) and longer (330 and 340 nm) wavelength groups, we found good correlation coefficient for the relationship between apoptosis and mutation at each wavelength group, that is, 0.9964 in the shorter wavelength group and 0.9129 in the longer wavelength group, respectively, although we found no relationship between apoptosis and mutation if not separated. This difference was reflected in the action spectra, *i.e.,* the curves for mutation were not parallel to those for apoptosis. It is possible that there is a tendency for apoptosis to be the response for damage due to 310- and 320-nm light, and for mutation to be the response for damage due to 330- and 340-nm light (Fig. 8 (C)). The balance between apoptosis and mutation seems to depend on whether there is a lack of DNA damage repair. When we used another NER-deficient strain, *mei-9*, the profile of apoptosis induced by UV differed from that in *mus201*. Although we have no data to explain why there was a difference between *mus201* and *mei-9*, the difference may be due to some native property of each strain, *i.e.,* it might be due to differences in the roles of the deficiencies in the repair pathway, since *mus201* is a homologue of XPG and *mei-9* is a homologue of XPF [48]. When we used *mei*^+^ as a wild-type strain, the profile of apoptosis was similar to that of the wild-type *mwh/flr* flies (data not shown). When we performed experiments using an NER-deficient strain (*mei-9*) and ATR-deficient strain (*mei-41*), apoptosis occurred in a different manner in each strain (Supplemental Fig. 5). In the *mei-9* strain, apoptosis increased continuously with irradiation at 310, 320, and 340 nm, but at 330 and 360 nm, it decreased after initially increasing. In contrast, in the *mei-41* strain, apoptosis increased, then decreased at all wavelengths tested, similar to the results of the wild-type *Drosophila*. These results are considered to reflect the different kinds of damage caused by the shorter and longer wavelengths, and appear to be in-line with those of our previous report, in which we showed that the mutagenic damage induced by UV light with a shorter wavelength differed from that with a longer wavelength [35]. Light at 310 to 320 nm induces DNA damage that is subject to NER. On the other hand, light at 330 to 360 nm causes damage that is recognized by a cell-cycle checkpoint, the ataxia-telangiectasia-mutated (ATM) and ataxia telangiectasia and Rad3-related (ATR) checkpoint, and the damage can be repaired by post-replication repair, but not by NER. Recently, we revealed that 365-nm light from a light-emitting diode caused oxidative damage that resulted in DNA recombination due to double-strand breaks, which should be repaired by the ATM pathway [49].

With X-ray irradiation, there was a correlation between apoptosis and somatic cell mutation (Fig. 9 (B)). Haynie and Bryant have reported that higher X-ray doses resulted in a decrease in the total number of cells in wing discs, while the total cell number did not change at lower X-ray doses [50], and they also observed pycnotic cells on wing discs at 12 h after X-ray irradiation. The pycnotic cells appear to be the same cells as the apoptotic cells seen in this study. In the wing-spot test, the number of mutant cells in a spot*, i.e.,* the spot size, reflects the number of cell divisions after mutagenic events [14]. In the experiment with X-ray irradiation, the spot size increased with increasing X-ray dose, and the number of AO-stained clusters showed a good correlation with the spot size (Fig. 12). Similarly, the spot size increased after MNU treatment (Fig. 10), however, in the UV exposure the spot size distribution unchanged even when mutation increased in parallel with the increase of UV dose (Fig. 11). These results suggest that the mutated cells proliferated and expanded to fill the space left by the cells that were removed by apoptosis after the treatment with MNU or X-ray irradiation.

**Fig. 14:**
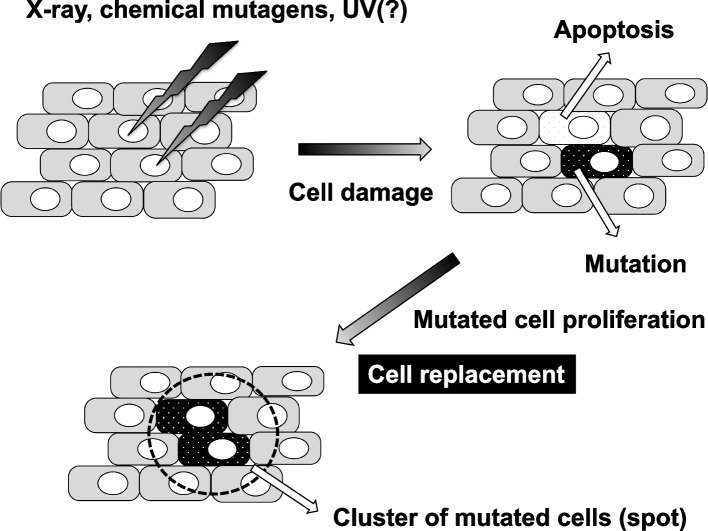
A schematic diagram showing the process from the induction of cell damage to the formation of a cluster of mutated cells (spot). After treatment with a mutagenic factor, such as X-ray irradiation, damaged cells are considered to undergo mutation or apoptosis. The mutated cells proliferate and fill the empty areas left by the removed apoptotic cells, forming a cluster of mutated cells, *i.e.,* a spot

The analysis of BrdU incorporation showed that cell proliferation was inhibited at 6 h, with peak inhibition at 12 h after X-ray irradiation, and that the cells started to proliferate again at 24 h post-irradiation (Fig. 13). This is in agreement with a report by Brodsky et al. [51], in which ionizing radiation stopped cell division through G2 arrest transiently. In addition, our data are supported by the notion proposed by Kondo [52] that cell replacement repair occurs in injured tissue. Ryoo et al*.* [53] and Martín et al*.* [54] showed that apoptotic cells could induce compensatory cell proliferation through signals produced by some genes, such as *wingless*. Therefore, we propose that apoptosis is correlated with the suppression of cell growth and the proliferation of mutated cells through the replacement of mutated cells in *Drosophila*, as shown in Fig. 14. Although they did not discuss which cells were involved in cell replacement repair in injured tissue, there is a possibility that the mutated cells receive signals to proliferate as a result of X-rays or MNU, but not UV. Further studies are needed to elucidate the mechanism of cell replacement induced by X-rays and MNU, but not by UV.

In *Drosophila*, apoptosis is necessary for normal development, and it is well-established that caspases play indispensable roles in developmentally regulated apoptotic events. To date, seven caspases that function in *Drosophila* development have been identified [55, 56]. Their functional properties correspond to those of some mammalian caspases. In the present study, we investigated the activity of DRIC (DEVDase), which corresponds to mammalian caspase-3, as an effector caspase (executioner caspase) [57], and IETDase, which was identified as the *Drosophila* homologue (DREDD) of the mammalian initiator caspase-8 [58]. Their activities did not change significantly with increasing apoptosis after X-ray or UV irradiation (data not shown). Although we were unable to identify which caspase(s) are involved in X-ray or UV-induced apoptosis in *Drosophila*, caspases other than those examined in this study might be required for cell damage-induced apoptosis. Recently, the function of p53 in DNA damage-induced apoptosis has been proved, as reviewed by Zhou [59]. Further studies are required to determine how caspases and their regulatory gene products are involved in cell damage-induced apoptosis and mutation in *Drosophila*.

## Conclusions

In this study, we showed that AO staining is one of the most suitable in situ methods for detecting apoptosis in *Drosophila* wing discs, because it enabled the continuous observation of time-dependent changes in apoptosis after treatment with mutagens. At non-toxic doses in wild-type *Drosophila*, apoptosis started to increase just after the treatment was stopped, peaked at 6 to 12 h, then decreased to levels close to those of the non-treated controls at 24 h. The intensity of apoptosis induced by mutagenic factors showed a good correlation with the mutagenicity detected in the wing-spot test at non-toxic, but mutagenic doses. From these results, DNA damage-induced apoptosis and mutation appear to be coordinated with each other. From the analysis of spot size and BrdU uptake, we propose that mutant spots on wings might be formed by the replacement of apoptotic cells with mutated cells after exposure to some mutagens, such as X-ray irradiation and MNU. In the case of UV light, the incidence of apoptosis was also correlated with mutation, but no expansion of mutated cells was observed. It remains to be investigated why these differences between mutagenic factors occur.

From our data, we consider that the induction of mutation, apoptosis, and/or cell growth varies in multi-cellular organisms depending on the type of the mutagens, and that their balance and coordination have an important function in cell and tissue maintenance for the survival of the organism.

## Supplementary Information

**Additional file 1.** Supplementary figures

**Additional file 2.** Supplementary tables

## Data Availability

Not applicable.

## Abbreviations

AO: Acridine orange
TUNEL: TdT-mediated dUTP-biotin nick end-labeling
UV: Ultraviolet
NDMA: *N*-Nitrosodimethylamine
NDEA: *N*-Nitrosodiethylamine
MNU: *N*-Methyl-*N*-nitrosourea
ENU: *N*-Ethyl-*N*-nitrosourea
BrdU: 5-Bromo-2’-deoxyuridine

## References

[1] Friedberg EC (2003). DNA damage and repair. Nature.

[2] Iyer RR, Pluciennik A, Burdett V, Modrich PL (2006). DNA mismatch repair: functions and mechanisms. Chem Rev.

[3] Hashimoto S, Anai H, Hanada K (2016). Mechanism of interstrand DNA crosslink repair and human disorders. Gene Environ.

[4] Kusakabe M, Onishi Y, Tada H, Kurihara F, Kusao K, Furukawa M, Iwai S, Yokoi M, Sakai W, Sugasawa K (2019). Mechanism and regulation of DNA damage recognition in nucleotide excision repair. Gene Environ.

[5] Lee T-H, Kang T-H (2019). DNA oxidation and excision repair pathways. Int J Mol Sci.

[6] Taylor RC, Cullen SP, Martin SJ (2008). Apoptosis: controlled demolition at the cellular level. Nat Rev Mol Cell Biol.

[7] Bosurgi L, Hughes LD, Rothlin CV, Ghosh S (2017). Death begets a new beginning. Immunol Rev.

[8] Song Y-H (2005). *Drosophila melanogaster*: a model for the study of DNA damage checkpoint response. Mol Cells.

[9] Yamaguchi M, Yoshida H (2018). *Drosophila* as a model organism. Adv Exp Med Biol.

[10] Staats S, Lüersen K, Wagner AE, Rimbach G (2018). *Drosophila melanogaster* as a versatile model organism in food and nutrition research. J Agrec Food Chem.

[11] Younes S, Al-Sulaiti S, Nasser EAA, Najjar H, Kamareddile L (2020). *Drosophila* as a model organism in host-pathogen interaction studies. Front Cell Infect Microbiol.

[12] Beira JV, Paro R (2016). The legacy of *Drosophila* imaginal discs. Chromosoma.

[13] Denton D, Aung-Htut MT, Kumar S (2013). Developmentally programmed cell death in *Drosophila*. Biochim Biophys Acta.

[14] Graf U, Würgler EF, Katz AJ, Frei H, Juon H, Hall CB, Kale PG (1984). Somatic mutation and recombination test in *Drosophila melanogaster*. Environ Mutagen.

[15] Kaina B (2003). DNA damage-triggered apoptosis: critical role of DNA repair, double-strand breaks, cell proliferation and signaling. Biochem Pharmacol.

[16] Roos WP, Kaina B (2006). DNA damage-induced cell death by apoptosis. Trends Mol Med.

[17] Baonza A, Tur-Gracia S, Pérez-Aguilera M, Estella C (2022). Regulation and coordination of the different DNA damage responses in *Drosophila*. Front Cell Dev Biol.

[18] Archana M, Yogesh TL, Kumaraswamy KL, Bastian (2013). Various methods available for detection of apoptotic cells- a review. Indian J Cancer..

[19] Majtnerová P, Roušar T (2018). An overview of apoptosis assays detecting DNA fragmentation. Mol Biol Rep.

[20] Kasibhatla S, Amarante-Mendes GP, Finucane D, Brunner T, Bossy-Wetzel E, Green DR (2006). Acridine orange/ethidium broimide (AO/EB) staining to detect apoptosis. CSH Protoc..

[21] Liu K, Liu P-C, Liu R, Wu X (2015). Dual AO/EB staining to detect apoptosis in osteosarcoma cells compared with flow cytometry. Med Sci Monit Basic Res.

[22] Arama E, Steller H (2006). Detection of apoptosis by terminal deoxynucleotidyl transferase-mediated dUTP nick-end labeling and acridine orange in Drosophila embryos and adult male gonads. Nat Protec.

[23] Boyd JB, Snyder RD, Harris PV, Presley JM, Boyd SF, Smith PD (1982). Identification of a second locus in *Drosophila melanogaster* required for excision repair. Genetics.

[24] Boyd JB, Golino MD, Setlow RB (1976). The *mei-9 *^*a*^ mutant of *Drosophila melanogaster* increases mutagen sensitivity and decreases excision repair. Genetics.

[25] Boyd JB, Setlow RB (1976). Characterization of postreplication repair in mutagen-sensitive strains of *Drosophila melanogaster*. Genetics.

[26] Hari KL, Santerre A, Sekelsky JJ, McKim KS, Boyd JB, Hawley RS (1995). The *mei-41* gene of *D. melanogaster* is a structural and functional homolog of the human ataxia telangiectasia gene. Cell..

[27] LaRocque JR, Jaklevic B, Su TT, Sekelsky J (2007). *Drosophila* ATR in double-strand break repair. Genetics.

[28] Wahl RC, Warner CK, Finnerty V, Rajagopalan KV (1982). *Drosophila melanogaster ma-l* mutants are defective in the sulfuration of desulfo Mo hydroxylases. J Biol Chem.

[29] Lindsley DL, Zimm GG (1992). The Genome of *Drosophila melanogaster*.

[30] Goto Y, Matsuda T, Ito K, Huh Nh, Thomale J, Rajewsky MF, Hayatsu H, Negishi T (1999). Mutagenicities of *N*- nitrosodimethylamine and *N*-nitrosodiethylamine in *Drosophila* and their relationship to the levels of *O*-alkyl adducts in DNA. Mutat Res..

[31] Hamatake Y, Morita A, Yuma Y, Okamoto K, Arimoto S, Suzuki T, Kasai H, Kawai K, Negishi T (2009). Hypersensitivity of a urate-null strain of *Drosophila melanogaster* to the toxic effects of environmental cigarette smoke. Gene Environ.

[32] Fujiwara M, Hamatake Y, Arimoto S, Okamoto K, Suzuki T, Negishi T (2011). Exposure to cigarette smoke increases urate level and decreases glutathione level in larval *Drosophila melanogaster*. Gene Environ.

[33] Uchiyama T, Koike R, Yuma Y, Okamoto K, Arimoto-Kobayashi S, Suzuki T, Negishi T (2016). Somatic-cell mutation induced by short exposures to cigarette smoke in urate-null, oxidative stress-sensitive *Drosophila*. Mutagenesis.

[34] Negishi T, Nagaoka C, Hayatsu H, Suzuki K, Hara T, Kubota M, Watanabe M, Hieda K (2001). Somatic-cell mutation induced by UVA and monochromatic UV radiation in repair-proficient and -deficient *Drosophila melanogaster*. Photochem Photobiol.

[35] Toyoshima M, Takinami S, Hieda K, Furusawa Y, Negishi T (2002). The involvement of cell cycle checkpoint-mutations in the mutagenesis induced in *Drosophila* by a longer wavelength light band of solar UV. Photochem Photobiol Sci.

[36] Negishi T, Kawai K, Arakawa R, Higashi S, Nakamura T, Watanabe M, Kasai H, Fujikawa K (2007). Increased levels of 8-hydroxy-2’-deoxyguanosine in *Drosophila* larval DNA after irradiation with 364-nm laser light but not with X-rays. Photochem Photobiol.

[37] Koike R, Uchiyama T, Arimoto-Kobayashi S, Okamoto K, Negihsi T (2018). Increase of somatic cell mutations in oxidative damage-sensitive *Drosophila*. Gene Environ.

[38] Frei H, Würgler FE (1988). Statistical methods to decide whether mutagenicity test data from *Drosophila* assays indicate a positive, negative, or inconclusive result. Mutat Res.

[39] Kastenbaum MA, Bowman KO (1970). Tables for determining the statistical significance of mutation frequencies. Mutat Res.

[40] Gavrieli Y, Sheman Y, Ben-Sasson SA (1992). Identification of programmed cell death *in situ* via specific labeling of nuclear DNA fragmentation. J Cell Biol.

[41] Fujikawa K, Hasegawa Y, Matsuzawa S, Fukunaga A, Itoh T, Kondo S (2000). Dose and dose-rate effects of X rays and fission neutrons on lymphocyte apoptosis in p53(+/+) and p53(-/-) mice. J Radiat Res.

[42] Wilson JM, Potten CS, Studzinski GP (1999). Morphological recognition of apoptotic cells. Apoptosis: A Practical Approach.

[43] Sarkissian T, Timmons A, Arya R, Abdelwahid E, White K (2014). Detecting apoptosis in *Drosophila* tissues and cells. Methods.

[44] Denton D, Kumar S (2015). Using the vital dye acridine orange to detect dying cells in *Drosophila*. Cold Spring Harb Protoc.

[45] Hirose F, Ohshima N, Shiraki M, Inoue YH, Taguchi O, Nishi Y, Matsukage A, Yamaguchi M (2001). Ectopic expression of DREF induces DNA synthesis, apoptosis, and unusual morphogenesis in the *Drosophila* eye imaginal disc: possible interaction with *Polycomb* and *trithorax* group proteins. Mol Cell Biol.

[46] Otsuki K, Hayashi Y, Kato M, Yoshida H, Yamaguchi M (2004). Characterization of dRFX2, a novel RFX family protein in *Drosophila*. Nucleic Acids Res.

[47] Takinami S, Mochizuki M, Hayatsu H, Nikaido O, Kubota M, Watanabe M, Hieda M, Hieda K, Negishi T (2000). Somatic cell mutation and photoproduct formation in Drosophila induced by monochromatic UV light in sunlight. Environ Toxicol..

[48] Sekelsky JJ, Hollis KJ, Eimerl AI, Burtis KC, Hawley RS (2000). Nucleotide excision repair endonuclease genes in *Drosophila melanogaster*. Mutat Res.

[49] Fang X, Ide N, Higashi S, Kamei Y, Toyooka T, Ibuki Y, Kawai K, Kasai H, Okamoto K, Arimoto-Kobayashi S, Negishi T (2014). Somatic cell mutations caused by 365 nm LED-UVA due to DNA double-strand breaks through oxidative damage. Photochem Photobiol Sci.

[50] Haynie JL, Bryant PJ (1977). The effects of X-rays on proliferation dynamics of cells in the imaginal wing disc of *Drosophila melanogaster*. Wilhelm Roux Arch Dev Biol.

[51] Brodsky MH, Sekelsky JJ, Tsang G, Hawley RS, Rubin GM (2000). *mus304* encodes a novel DNA damage checkpoint protein required during *Drosophila* development. Genes Dev.

[52] Kondo S (1998). Apoptotic repair of genotoxic tissue damage and the role of p53 gene. Mutat Res.

[53] Ryoo HD, Gorenc T, Steller H (2004). Apoptotic cells can induce compensatory cell proliferation through the JNK and the wingless signaling pathways. Develop Cell.

[54] Martín FA, Peréz-Garijo A, Morata G (2009). Apoptosis in *Drosophila*: compensatory proliferation and undead cells. Int J Dev Biol.

[55] Kumar S, Doumani J (2000). The fly caspase. Cell Death Differ.

[56] Kumar S (2007). Caspase function in programmed cell death. Cell Death Differ.

[57] Fraser AG, Evan GI (1977). Identification of a *Drosophila melanogaster* ICE/CED-3-related protease, driCE. EMBO J.

[58] Chen P, Rodriguez A, Erskine R, Thach T, Abrams JM (1998). *Dredd*, a novel effector of the apoptosis activators *reaper*, *grim*, and *hid* in *Drosophila*. Dev Biol.

[59] Zhou L (2019). P53 and apoptosis in the *Drosophila* model. Adv Exp Med Biol.

