# Supplementary figures and images for: Mutation and apoptosis are well-coordinated for protecting against DNA damage-inducing toxicity in Drosophila
Source: Genes Environ. 2023 Mar 23;45:11. doi: 10.1186/s41021-023-00267-4 (PMC10035180; doi:10.1186/s41021-023-00267-4)

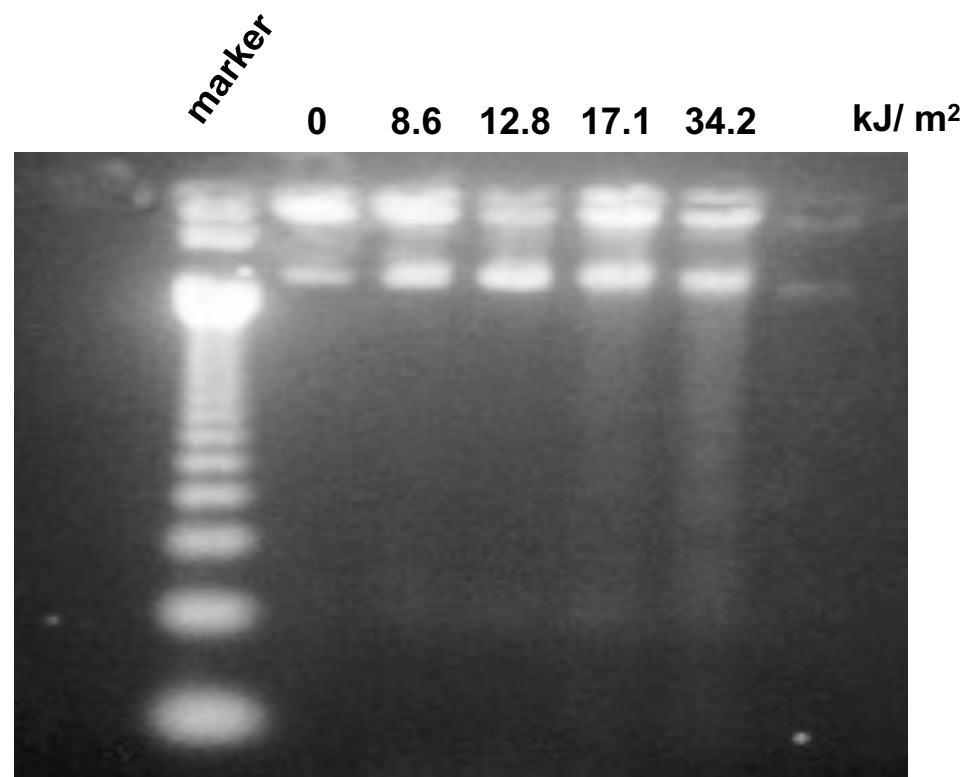

**(A) Paraffin-embedded wing disc**

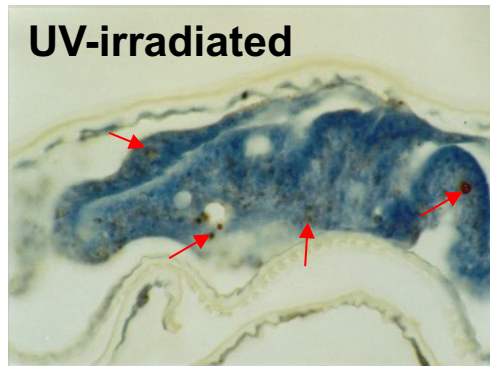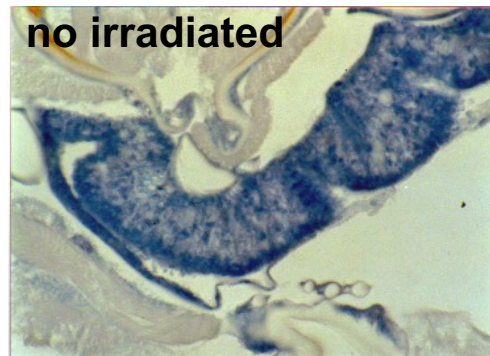

**(B) Dissected wing disc**

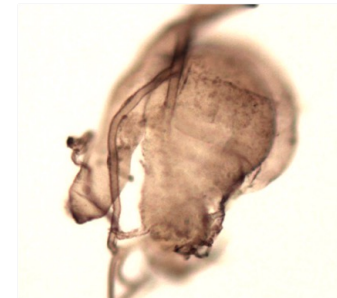

**0 h**

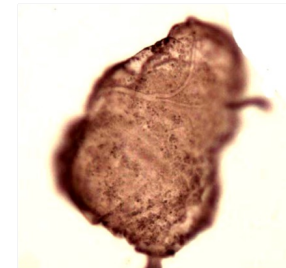

**12 h**

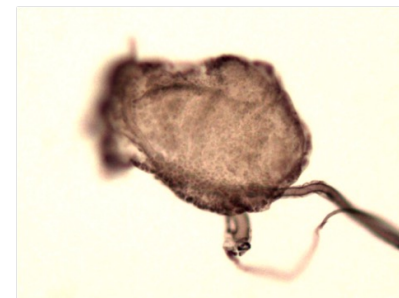

**24 h**

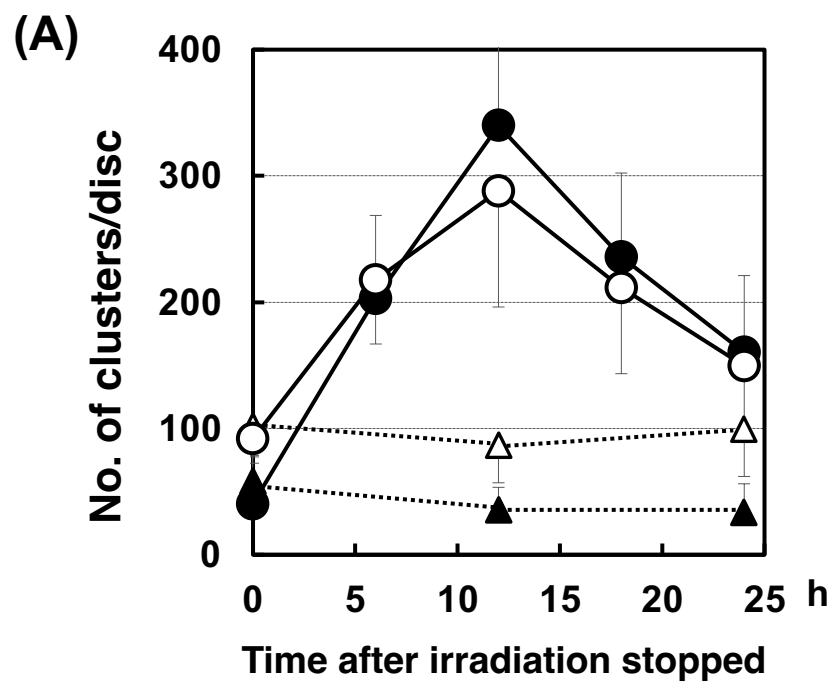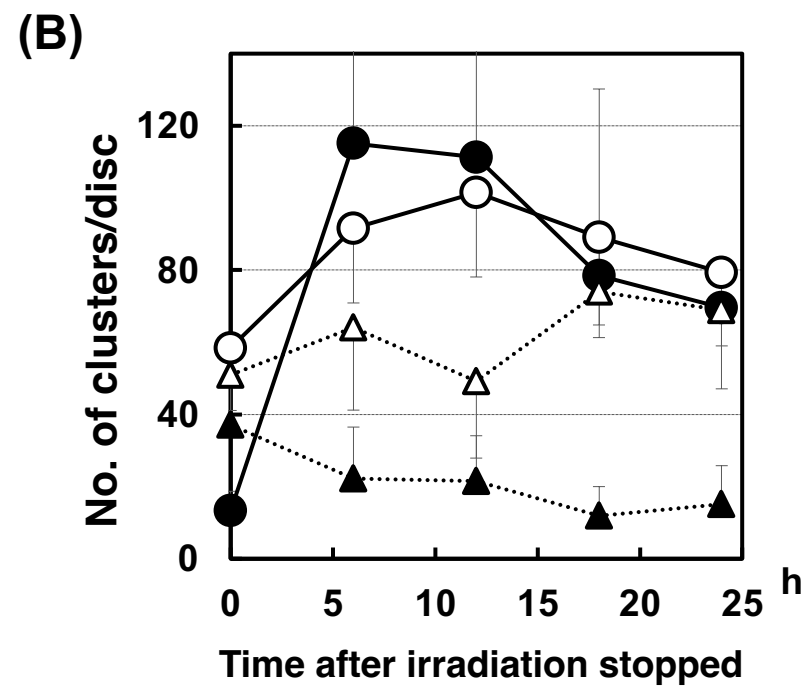

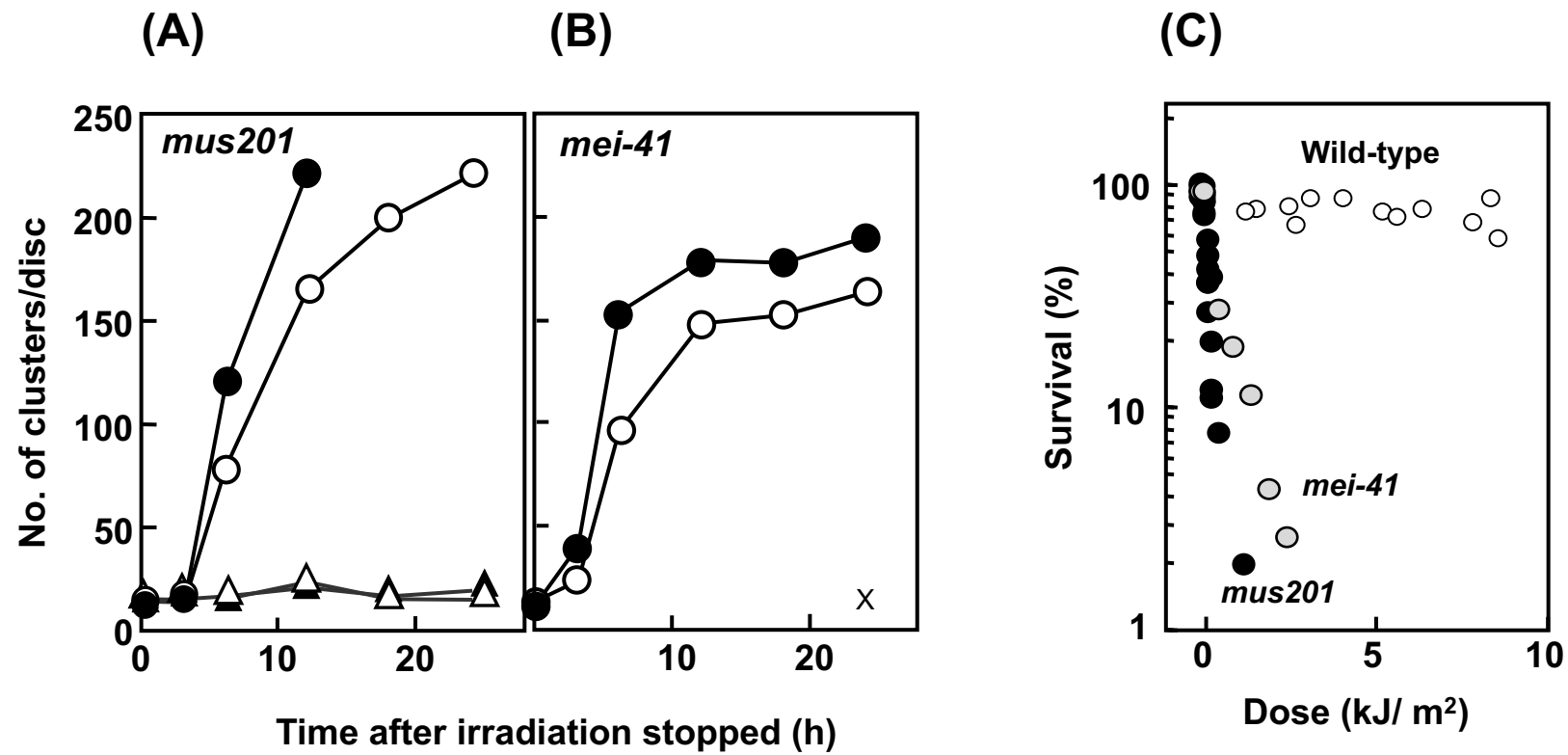

Suppl. Figure 4

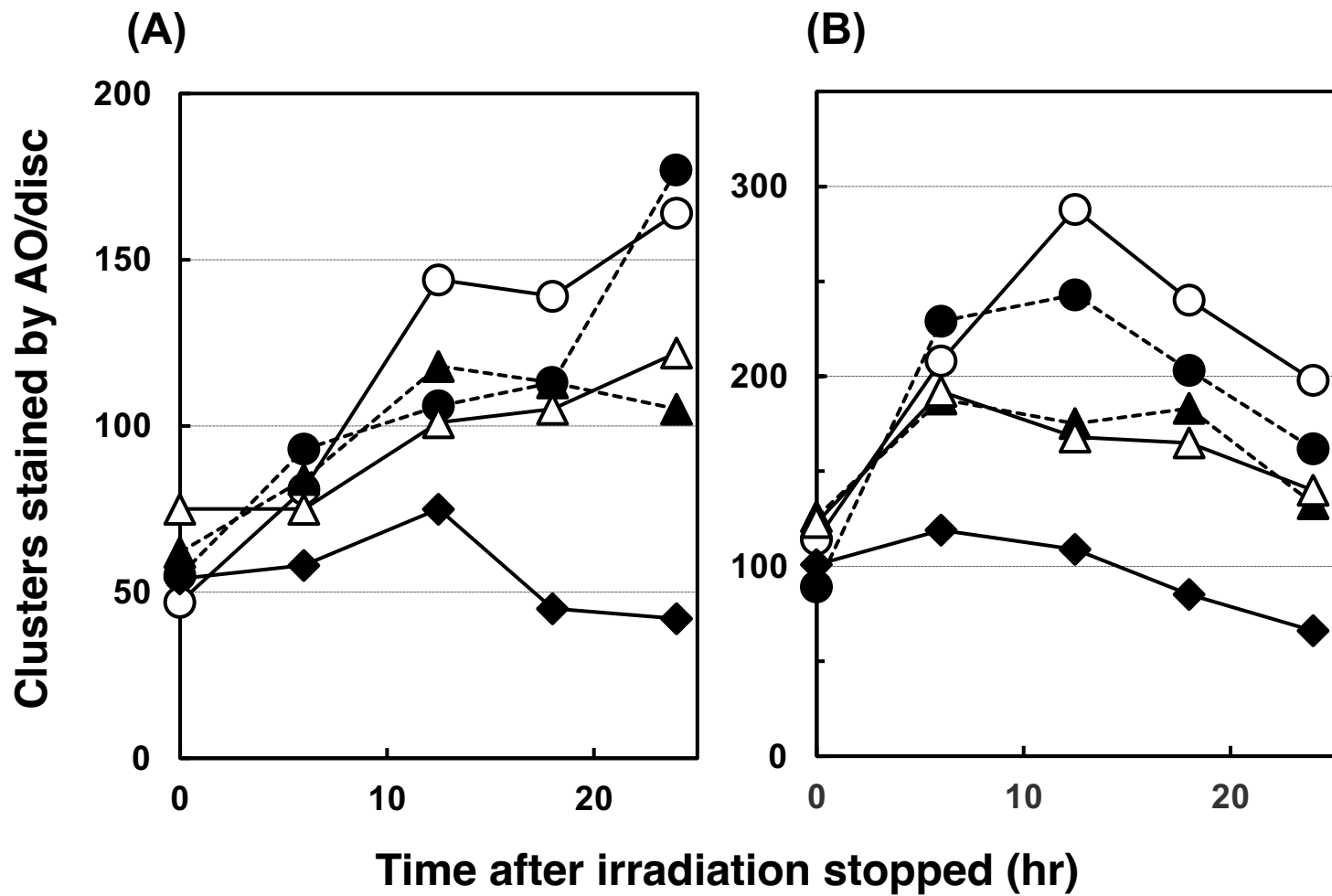

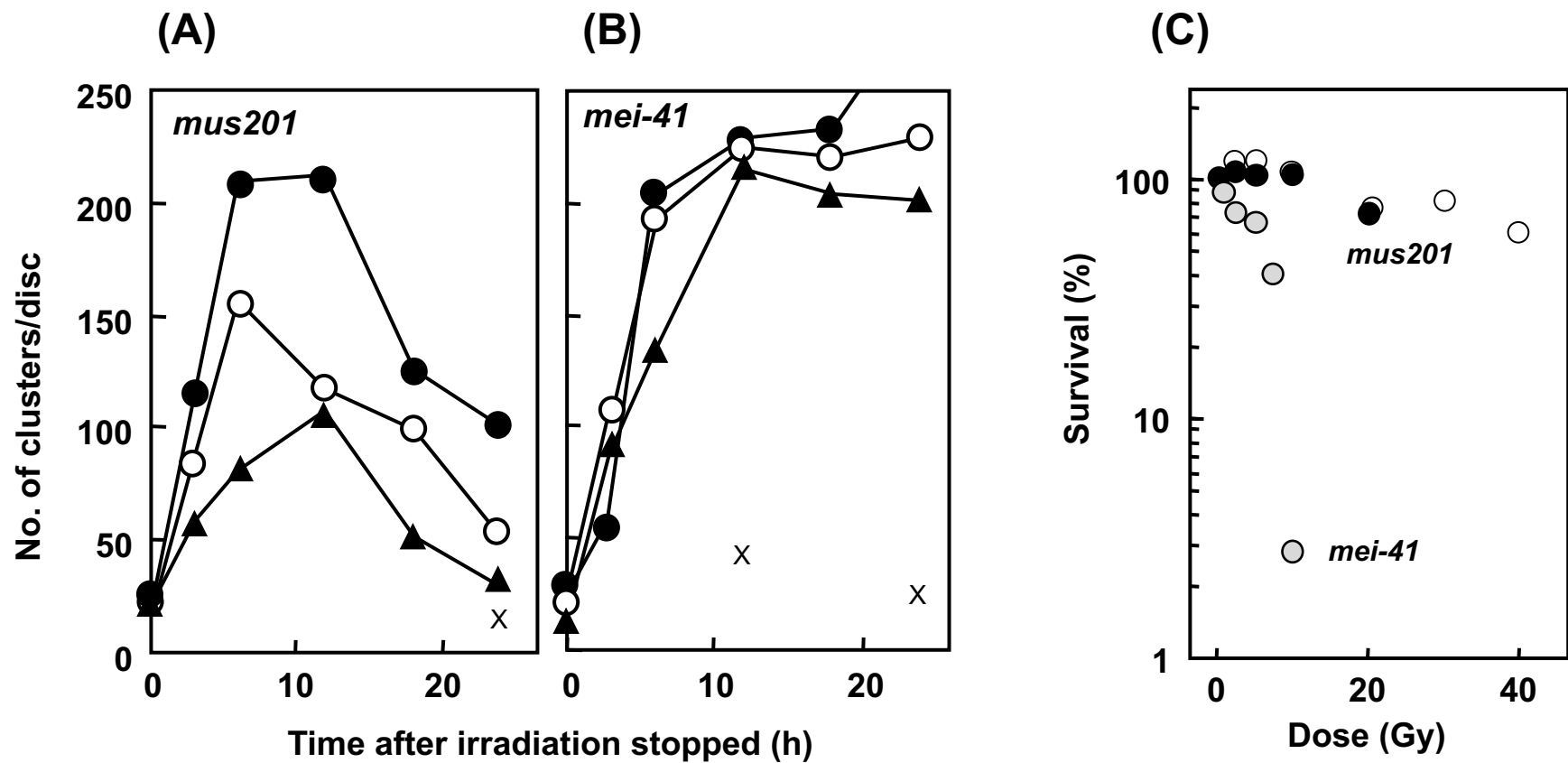

Suppl. Figure 6

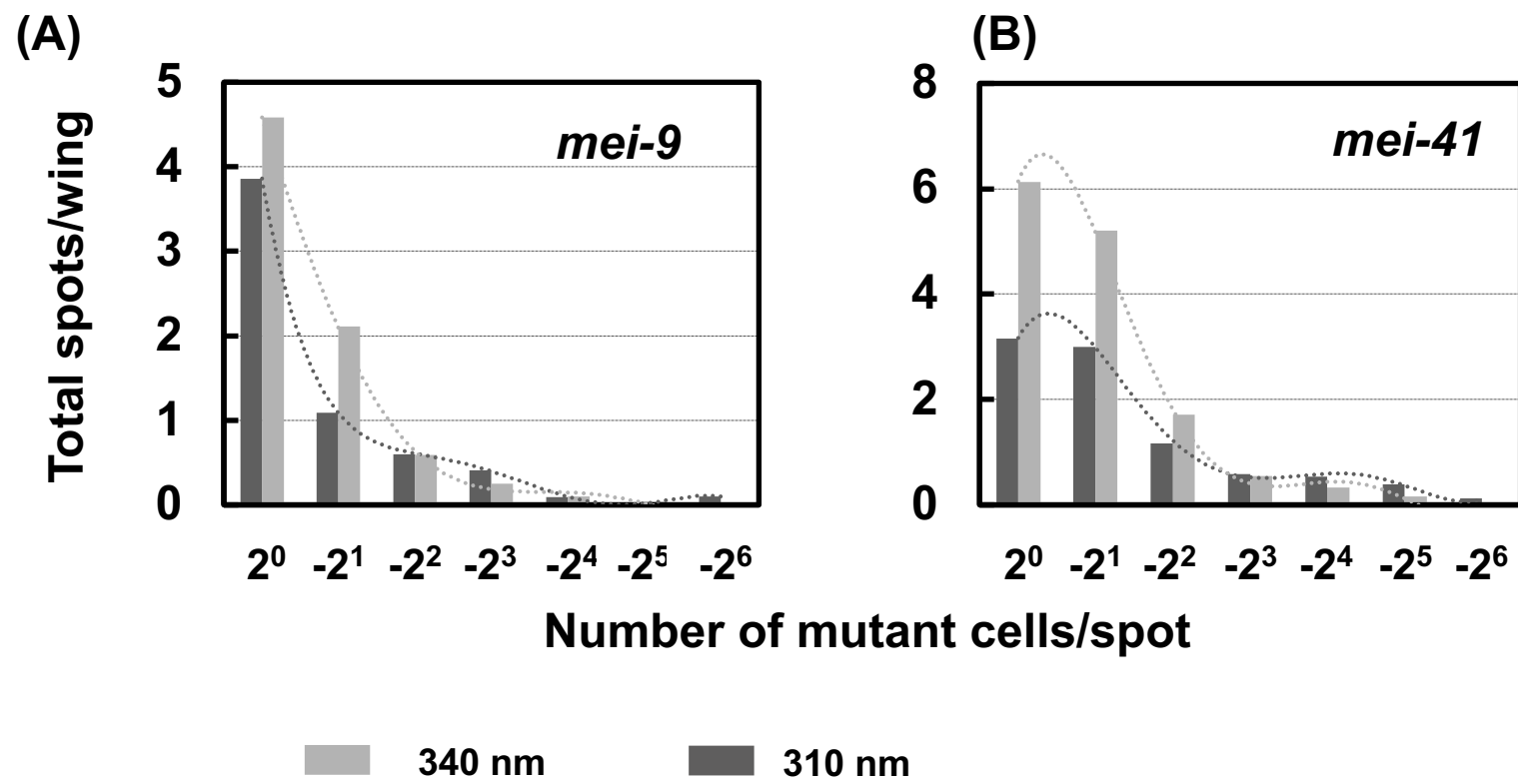

Supplement: Supplementary file 1 — Additional file 1. Supplementary figures [file 41021_2023_267_MOESM1_ESM.pdf]
