# Supplementary material for: Mutation and apoptosis are well-coordinated for protecting against DNA damage-inducing toxicity in Drosophila
Source: Genes Environ. 2023 Mar 23;45:11. doi: 10.1186/s41021-023-00267-4 (PMC10035180; doi:10.1186/s41021-023-00267-4)
Supplement: Supplementary file 2 — Additional file 2. Supplementary tables [file 41021_2023_267_MOESM2_ESM.docx]

Supplementary Table 1 The profile of apoptosis induction after X-ray irradiation in wild-type *Drosophila* (*mwh/flr*)

| Dose  (Gy) | Time post-irradiation | | | | |
| --- | --- | --- | --- | --- | --- |
|  | 0 h | 6 h | 12 h | 18 h | 24 h |
|  | The number of clusters/wing disc | | | | |
| TUNEL assay | | | | | |
| 0 | 101.7 ± 23.0 |  | 86.6 ± 29.6 |  | 99.0 ± 37.0 |
| 10 | 91.9 ± 19.4 | 217.8 ± 50.9 | 288.2 ± 92.0 | 211.3 ± 67.9 | 149.5 ± 50.9 |
| AO staining | | | | | |
| 0 | 58.2 ± 19.3 |  | 35.2 ± 18.2 |  | 33.2 ± 23.0 |
| 10 | 40.4 ± 20.9 | 203.0 ± 65.7 | 339.8 ±159.6 | 236.0 ± 66.3 | 160.3 ± 60.8 |

Supplementary Table 2 The profile of apoptosis induction after 310-nm monochromatic UV-light irradiation in wild-type *Drosophila* (*mwh/flr*)

| Dose  (kJ/m^2^) | Time post-irradiation | | | | |
| --- | --- | --- | --- | --- | --- |
|  | 0 h | 6 h | 12 h | 18 h | 24 h |
|  | The number of clusters/wing disc | | | | |
| TUNEL assay | | | | | |
| 0 | 51.0 ± 17.5 | 64.1 ± 22.9 | 49.2 ± 21.3 | 74.0 ± 09.2 | 68.8 ± 21.7 |
| 20 | 58.5 ± 17.4 | 91.5 ± 20.6 | 101.5 ± 23.4 | 89.1 ± 27.8 | 79.4 ± 20.4 |
| AO staining | | | | | |
| 0 | 37.1 ± 17.1 | 22.3 ± 14.2 | 21.6 ± 12.5 | 11.9 ± 08.1 | 15.2 ± 10.6 |
| 20 | 13.3 ± 05.4 | 115.1 ± 51.5 | 111.2 ± 39.0 | 78.5 ± 51.7 | 69.6 ± 27.4 |
